# Cognitive domains affected post‐COVID‐19; a systematic review and meta‐analysis

**DOI:** 10.1111/ene.16181

**Published:** 2024-02-20

**Authors:** Jack B. Fanshawe, Brendan F. Sargent, James B. Badenoch, Aman Saini, Cameron J. Watson, Aleksandra Pokrovskaya, Daruj Aniwattanapong, Isabella Conti, Charles Nye, Ella Burchill, Zain U. Hussain, Khanafi Said, Elinda Kuhoga, Kukatharmini Tharmaratnam, Sophie Pendered, Bernard Mbwele, Maxime Taquet, Greta K. Wood, Jonathan P. Rogers, Adam Hampshire, Alan Carson, Anthony S. David, Benedict D. Michael, Timothy R. Nicholson, Stella‐Maria Paddick, Charles E. Leek

**Affiliations:** ^1^ Department of Psychiatry University of Oxford Oxford UK; ^2^ Oxford Health NHS Foundation Trust Oxford UK; ^3^ Department of Clinical Infection, Microbiology and Immunology, Institute of Infection, Veterinary and Ecological Sciences University of Liverpool Liverpool UK; ^4^ Barts Health NHS Trust London UK; ^5^ Preventive Neurology Unit Queen Mary University of London London UK; ^6^ School of Life and Medical Sciences University College London London UK; ^7^ Institute of Psychiatry, Psychology and Neuroscience King's College London London UK; ^8^ South London and Maudsley NHS Foundation Trust London UK; ^9^ Department of Brain Sciences Imperial College London London UK; ^10^ Department of Psychiatry King Chulalongkorn Memorial Hospital Bangkok Thailand; ^11^ Gloucestershire Hospitals NHS Foundation Trust Gloucester UK; ^12^ Division of Psychiatry University College London London UK; ^13^ NHS Greater Glasgow and Clyde Glasgow UK; ^14^ Edinburgh Medical School University of Edinburgh Edinburgh UK; ^15^ Mbeya College of Health and Allied Sciences University of Dar es Salaam Mbeya Tanzania; ^16^ Centre for Clinical Brain Sciences University of Edinburgh Edinburgh UK; ^17^ NIHR Health Protection Research Unit in Emerging and Zoonotic Infections at University of Liverpool Liverpool UK; ^18^ Walton Centre NHS Foundation Trust Liverpool UK; ^19^ Translational and Clinical Research Institute Newcastle University Newcastle upon Tyne UK; ^20^ Gateshead Health NHS Foundation Trust Gateshead UK; ^21^ Department of Psychology University of Liverpool Liverpool UK

**Keywords:** attention, cognition, cognitive impairment, COVID‐19, executive function, language, learning, memory

## Abstract

**Background and purpose:**

This review aims to characterize the pattern of post‐COVID‐19 cognitive impairment, allowing better prediction of impact on daily function to inform clinical management and rehabilitation.

**Methods:**

A systematic review and meta‐analysis of neurocognitive sequelae following COVID‐19 was conducted, following PRISMA‐S guidelines. Studies were included if they reported domain‐specific cognitive assessment in patients with COVID‐19 at >4 weeks post‐infection. Studies were deemed high‐quality if they had >40 participants, utilized healthy controls, had low attrition rates and mitigated for confounders.

**Results:**

Five of the seven primary Diagnostic and Statistical Manual of Mental Disorders (DSM‐5) cognitive domains were assessed by enough high‐quality studies to facilitate meta‐analysis. Medium effect sizes indicating impairment in patients post‐COVID‐19 versus controls were seen across executive function (standardised mean difference (SMD) −0.45), learning and memory (SMD −0.55), complex attention (SMD −0.54) and language (SMD −0.54), with perceptual motor function appearing to be impacted to a greater degree (SMD −0.70). A narrative synthesis of the 56 low‐quality studies also suggested no obvious pattern of impairment.

**Conclusions:**

This review found moderate impairments across multiple domains of cognition in patients post‐COVID‐19, with no specific pattern. The reported literature was significantly heterogeneous, with a wide variety of cognitive tasks, small sample sizes and disparate initial disease severities limiting interpretability. The finding of consistent impairment across a range of cognitive tasks suggests broad, as opposed to domain‐specific, brain dysfunction. Future studies should utilize a harmonized test battery to facilitate inter‐study comparisons, whilst also accounting for the interactions between COVID‐19, neurological sequelae and mental health, the interplay between which might explain cognitive impairment.

## INTRODUCTION

As seen in previous coronavirus epidemics [1], a significant proportion of patients infected with SARS‐CoV‐2 develop cognitive (neuropsychological) sequelae from the infection [2], and a significant proportion of these persist long term [3]. Post‐COVID‐19 condition is defined by National Institutes of Health guidance as symptoms present 3 months from the onset of COVID‐19 with symptoms that have lasted at least 2 months and cannot be explained by an alternative diagnosis [4, 5]. Cognitive impairment appears to be common after recovering from COVID‐19 disease, with an estimated 18%–36% of people affected depending on whether subjective or objective measures are used [6], and 32% of patients reporting subjective ‘brain fog’ over 3 months after the initial infection [7].

Meta‐analysis demonstrates a prevalence of objectively measured global cognitive impairment of 22% at 12 weeks or more following COVID‐19 infection [6], compared to infected controls, and similar persistent cognitive impairment can be seen in formal testing even in those without persistent COVID‐19 symptomology [8]. Early reports suggested that COVID‐19 patients suffered from a dysexecutive syndrome during acute infection [9]; however, a detailed, domain‐specific phenotype of cognitive impairment occurring in individuals in this post‐acute phase is yet to be established [10].

An acute‐phase dysexecutive syndrome may suggest frontal lobe pathology [11, 12], but the neurobiological basis of post‐COVID‐19 cognitive impairment remains unclear, with multiple and multifactorial aetiologies being proposed [13, 15, 16, 17, 18]. Likely mediators include the well‐established cognitive sequelae of intensive care unit (ICU) admissions [19], acute respiratory distress syndrome and delirium [12, 20]. The medium‐ and long‐term cognitive symptoms experienced by those with asymptomatic or mild effects from the initial infection suggest a separate aetiology to those seen in patients with more direct effects such as stroke or encephalitis [21]. Although there is literature on how general severity of COVID‐19 illness may affect cognitive symptoms, fewer studies have explored how the presence of neurological sequelae in this context might affect outcomes.

Elucidating the pattern of cognitive impairment is key to understanding this pathophysiology, improving diagnosis and formulating management options for a condition that is likely to have an impact on quality of life, economic output and societal engagement [22, 23].

The aims of this review were therefore (1) to identify which primary cognitive domains are impaired in this population and, if present, whether these change over time, (2) to identify which neuropsychological tasks were used and how impairment was defined and (3) to establish whether any demographic or clinical factors predict the presence, and/or severity, of this impairment.

## METHODS

### Protocol and registration

This systematic review was conducted in line with the PRISMA‐S guidelines [24, 25]. The protocol was prospectively published on the PROSPERO database (CRD42022318721).

### Eligibility criteria

Prospective or retrospective cross‐sectional, case–control, case series or cohort studies were all considered for inclusion. Studies were included if they reported primary cognitive domain assessments in patients aged ≥18 years with a history of World Health Organization (WHO) criteria ‘confirmed’ (as indicated by polymerase chain reaction or antibody assays, of blood, bronchoalveolar lavage, cerebrospinal fluid or oronasopharyngeal swabs) or clinically ‘suspected’ SARS‐CoV‐2 infection at least 28 days from the acute infection or symptom onset. Only studies of previously validated cognitive assessments were included. Studies were excluded if they had five or fewer patients.

There were no further limits on patient cohorts, in terms of clinical characteristics, demographics, comorbidities or treatments. For a global perspective, articles in any language were included where translation was feasible within the author team (one included study was translated from Russian).

### Search strategy

Multiple databases were searched for potentially relevant records. These included MEDLINE (via PubMed), Embase (via OvidSP) and PsycINFO (via OvidSP). Grey literature was searched through abstracts on Embase and ongoing studies via the WHO International Clinical Trials Registry Platform. Lastly, a manual reference review of included articles and identified reviews was conducted (full search strategy in Data S1, Supplementary Materials 1). The search was last updated on 25 August 2022.

### Study selection process

Study selection was conducted utilizing Rayyan AI software [26]. After deduplication, studies were independently screened by two reviewers (JBF, BFS, JBB, AS, AP, BM, DA, IC, CN, EB, ZH, KS, EK) against the inclusion criteria. Discrepancies were discussed until 100% agreement was reached with a third reviewer acting as adjudicator (CL, SMP).

### Data extraction process

Data extraction was conducted by two independent reviewers (AS, JBB, AP, DA, IC, CN, EB, ZH, BS, JBF) onto a predefined and piloted extraction sheet. Discrepancies were discussed until 100% agreement was reached with a third author acting as adjudicator. Data were extracted on a study level basis and as direct outcome measures. The primary outcome measures extracted included the number of patients with and without significant levels of impairment in assessed domains and raw outcomes for each cognitive test in the form of means and standard deviations for patients and healthy controls. Patient, disease and treatment factors for prognostic factors were also established if available in the form of odds ratios, hazard ratios, correlation coefficients or means differences.

### Strategy for data synthesis

The primary and secondary cognitive domains measured by each test were mapped onto the Diagnostic and Statistical Manual of Mental Disorders (DSM‐5) framework [27]; initially this was done individually by two academics with expertise in neuropsychological testing (SMP and CL) with disagreements and final consensus on all tasks discussed with the wider COVID‐CNS neurocognitive working group (BM, ASD, AH, TN, AC) at regular meetings. The primary domains were executive function, learning and memory, language, visuospatial cognition, perceptual motor function, complex attention and social cognition.

Due to significant heterogeneity of patient characteristics and outcome measures in identified studies, a meta‐analysis was only conducted on the studies that met all of the following quality criteria: (1) combined *n* of post‐COVID patients and healthy controls >40; (2) attrition rates <20%; (3) adjustment for confounders by matched cohorts, stratification or appropriate adjustment in analysis. A meta‐analysis was performed when more than two studies (1) reported standardised mean differences (SMDs) for assessment of primary domain impairment or (2) matched prognostic exposures and outcome pairs. The meta‐analysis was conducted using R version 4.3.1 and the metafor package version 4.2.0 [28]. The a priori threshold for statistical significance was set to *p* < 0.05. An SMD of 0.15 was considered to be a small effect size, 0.40 medium and 0.75 large, as these thresholds have previously been established as the 25th, 50th and 75th percentile ranks for research summarized meta‐analyses [29]. A generic inverse variance approach was chosen to accommodate the range of effect size measures, whilst a random‐effects model was used to mitigate between‐study heterogeneity. Between‐study variance was estimated using the restricted maximum likelihood estimator method [16, 17], and confidence intervals were calculated based on a standard normal distribution. The proportion of the variation in effect sizes that was due to between‐study variability was quantified using the *I* ^2^ test. Assessment of reporting bias was conducted using a funnel plot for each exposure−outcome pair. Results were generated for the exposure (post‐COVID patients vs. healthy controls) and each outcome (primary domains as outlined above).

A narrative synthesis of other results was performed. This included a per‐outcome predetermined analysis using a range of results through descriptive methods. For the narrative synthesis impairment was defined as (1) a statistically significant difference in test scores between post‐COVID‐19 groups and controls or (2) a statistically significant difference in test scores from predefined adjusted norms. The outcome reported for this synthesis was presence of impairment, percentage of patients impaired (trichotomized into 1%–25%, 26%–50% and >50%) and severity of impairment if available. For both the meta‐analysis and narrative synthesis, when multiple tests were reported for a domain by a paper, priority was given to the test that matched the primary domain most closely.

### Risk of bias and strength of evidence assessment

Individual study quality assessment was conducted independently by two reviewers (BS, AS, AP, DA, JBB, IC, EB, JBF) with a third author acting as adjudicator if disagreement occurred. Risk of bias assessment was assessed at the study level and utilized the Joanna Briggs Institute tools for each relevant study design (Figure S5) [30, 31]. GRADE and QUIPS methodologies were used to identify bias and strength of evidence for prognostic factors' impact on domain‐specific cognitive outcomes (Figure S6 and Table S3) [14, 32].

## RESULTS

A total of 10,984 articles were screened for inclusion with 10,918 excluded via title, abstract and full text screening (Figure S1). A final 66 studies were included, with 6311 patients across studies, conducted in 16 different countries across four continents (Table S2). Of these countries 13 are classed as high‐income economies and three as middle‐income economies by the World Bank Criteria 2022, with a preponderance towards western European countries [33]. Ten studies met high‐quality criteria for inclusion in a meta‐analysis of standardized mean differences. A heterogeneous set of 134 different named cognitive tasks was used across all studies. Thirty‐three studies reported proportions of patients impaired in at least one domain, with the remaining either describing group‐wise differences or not providing detailed breakdowns (Figure S2 and Table S4).

### Executive function

Executive function was tested by 56 studies, making it the most frequently tested primary cognitive domain in this systematic review and meta‐analysis. The most frequently used tasks were the Trail Making Test B (*n* = 31), the Stroop Interference Test (*n* = 21) and Frontal Assessment Battery (*n* = 11).

**FIGURE 1:**
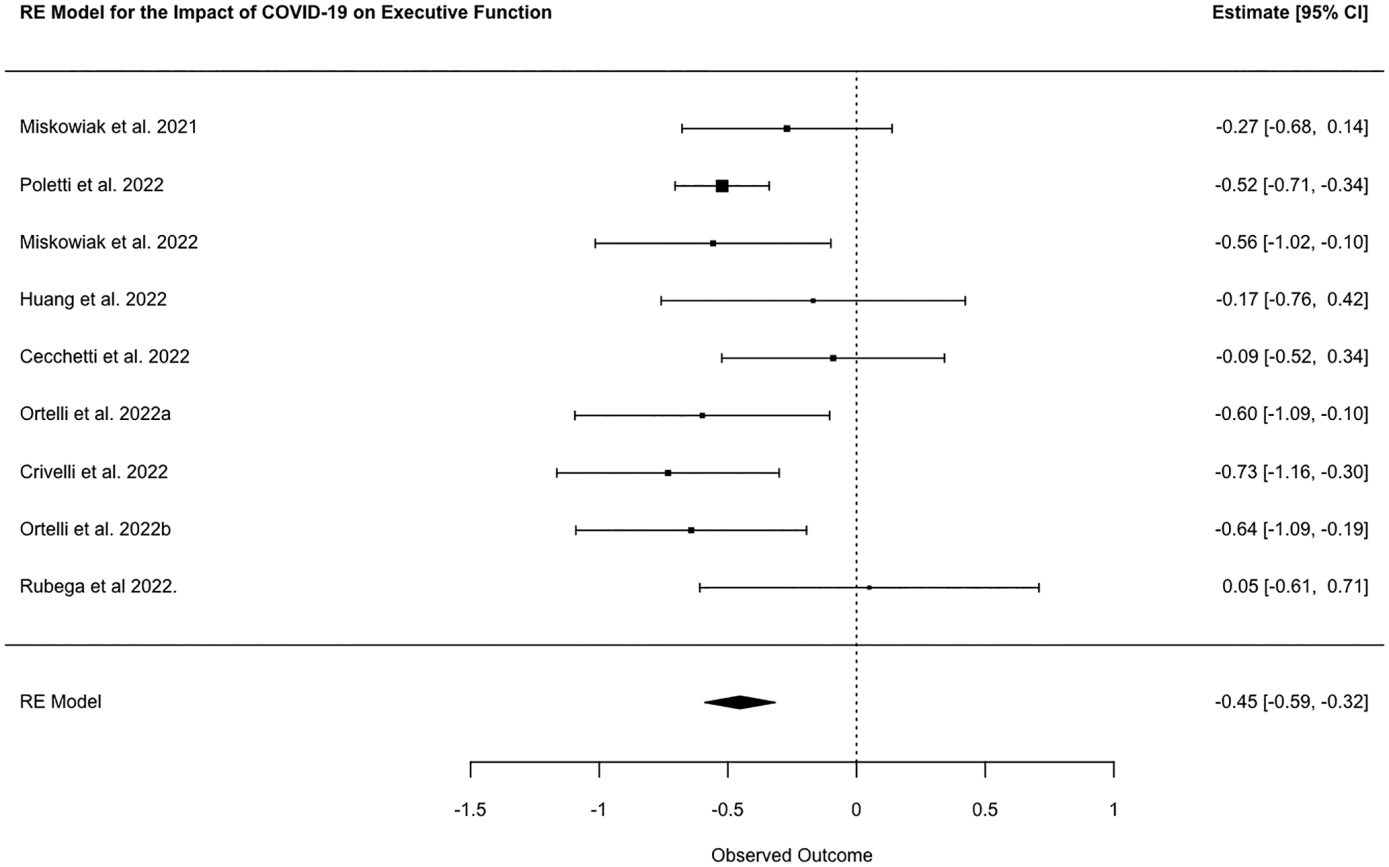
Forest plot of meta‐analysed SMDs for executive function test scores of patients post‐COVID‐19 disease and healthy controls.

A meta‐analysis of nine studies showed a combined SMD between cases and controls of −0.45 (95% confidence interval [CI] −0.59, −0.32, *N* = 1136, *p* ≤ 0.0001; *I* ^2^ 9.1%; Figure 1). Of the low‐quality studies (*k* = 33) that reported data for tests of executive function, seven reported a group level impairment between post‐COVID patients and either controls or normalized data whilst four reported no group level impairment. Of the 22 studies that reported the percentage of patients with impairments 10 reported 1%–25% of patients were impaired, nine reported 26%–50% of patients were impaired and three reported >50% of patients were impaired.

The secondary domain most frequently tested and most frequently impaired was working memory, followed by planning/reasoning/decision making. Typically, if multiple secondary domains were captured, there was impairment consistently across these secondary domains. Notable exceptions to this were two studies which both found impairment on the Wisconsin Card Sorting Test (10.2%–53%) and on the Stroop Test (10.2%–16%) but not on the Wechsler Adult Intelligence Scale Fourth Edition (WAIS‐IV) Similarities subtest [79, 105].

### Learning and memory

**FIGURE 2:**
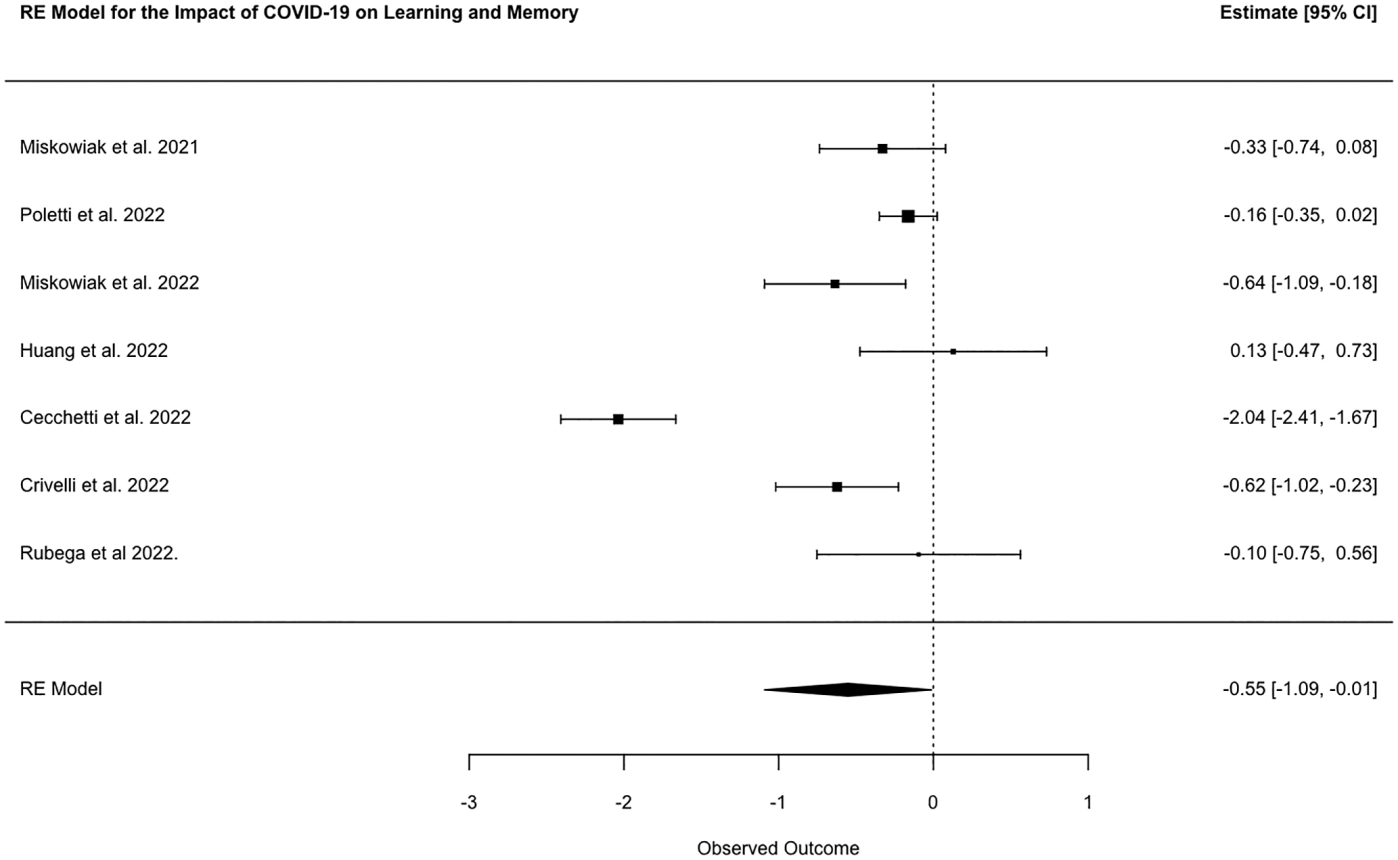
Forest plot of meta‐analysed SMDs for learning and memory test scores of patients post‐COVID‐19 disease and healthy controls.

Learning and memory was tested by 45 studies. The most frequently reported tests were Digit Span Forwards (*n* = 25), Digit Span Backwards (*n* = 23) and Rey Auditory Verbal Learning Test (*n* = 13). A meta‐analysis of seven studies showed a combined SMD between cases and controls of −0.55 (95% CI −1.09, −0.01, *N* = 949, *p* = 0.045; *I* ^2^ 92.62%; Figure 2).

Of the low‐quality studies (*k* = 34) that reported data for tests of learning and memory, four reported a group level impairment between post‐COVID patients and either controls or normalized data whilst three reported no group level impairment. Of the 27 studies that reported the percentage of patients with impairments 11 reported 1%–25% of patients were impaired, 13 reported 26%–50% of patients were impaired, and three reported >50% of patients were impaired. Most of these studies used tasks probing the secondary domains of long‐term memory, short‐term memory and verbal episodic memory. In papers that captured multiple secondary domains of learning and memory, no specific impairment was found.

### Perceptual motor function

**FIGURE 3:**
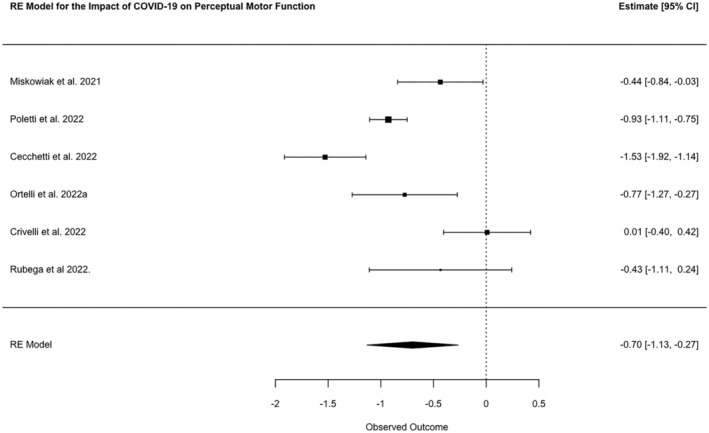
Forest plot of meta‐analysed SMDs for perceptual motor function test scores of patients post‐COVID‐19 disease and healthy controls.

Perceptual motor function was assessed by 37 studies, and the most frequently used tasks for this domain were the Trail Making Test A (*n* = 25) and Rey Figure Copy (*n* = 14). A meta‐analysis of six studies showed a combined SMD between cases and controls of −0.67 (95% CI −1.13, −0.27, *N* = 915, *p* = 0.0015; *I* ^2^ 87.08%; Figure 3).

Of the low‐quality studies (*k* = 25) that reported data for tests of language, two reported a group level impairment between post‐COVID patients and either controls or normalized data whilst four reported no group level impairment. Of the 19 studies that reported the percentage of patients with impairments, 11 reported 1%–25% of patients were impaired, six reported 26%–50% of patients were impaired, and two reported >50% of patients were impaired. Secondary domains were examined consistently across studies. Twenty‐one papers tested perceptual motor coordination, 19 tested visual perception/organization and 15 tested visuoconstructional reasoning; the studies that captured multiple secondary domains demonstrated uniform impairment across these.

### Language

**FIGURE 4:**
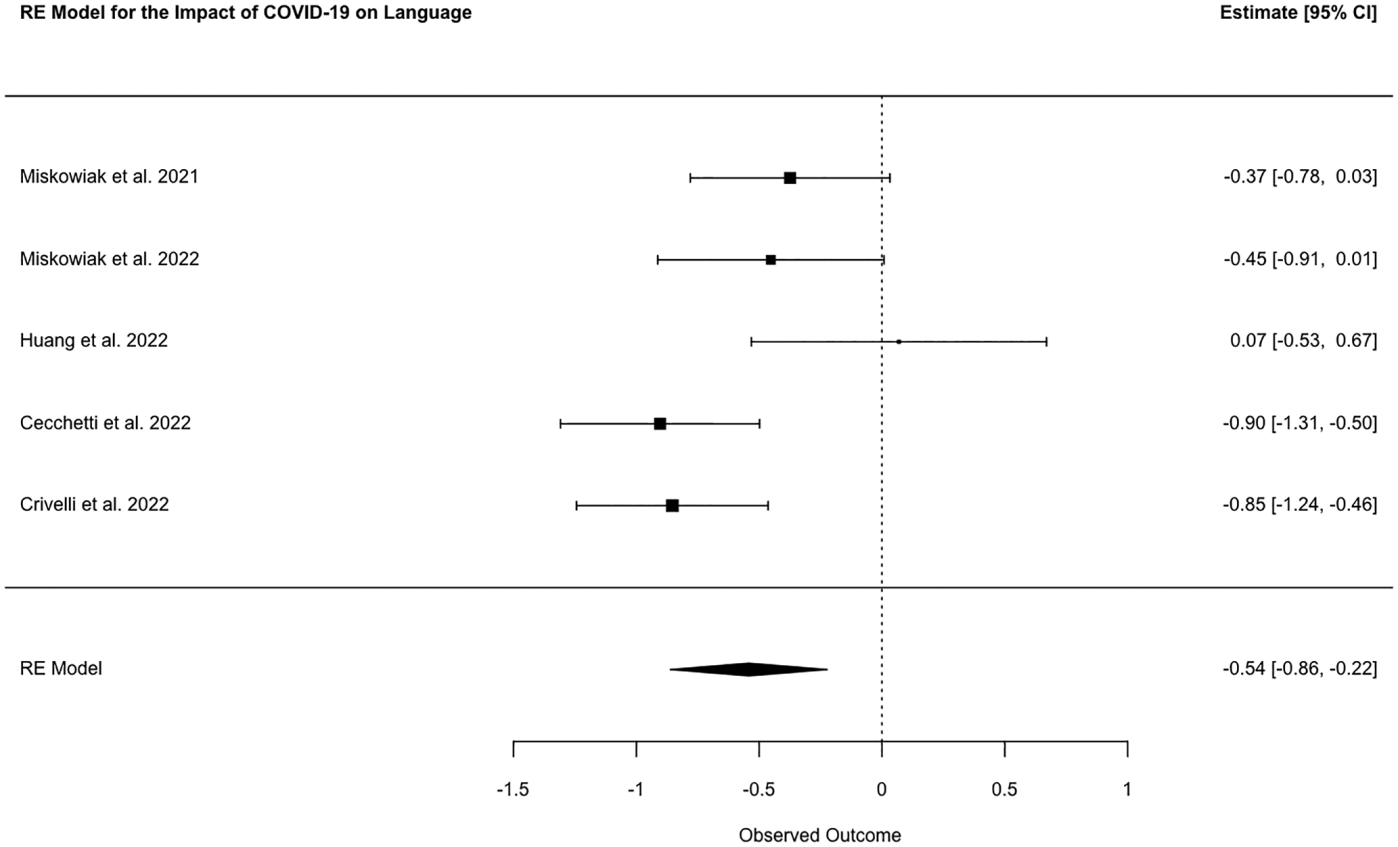
Forest plot of meta‐analysed SMDs for language test scores of patients post‐COVID‐19 disease and healthy controls.

Tests assessing the language domain were utilized by 35 studies. The Phonemic and Category Fluency Test (*n* = 11), Semantic Verbal Fluency Test (*n* = 11) and Controlled Oral Word Association Test (*n* = 6) were the most frequently used tasks. A meta‐analysis of five studies showed a combined SMD between cases and controls of −0.54 (95% CI −0.86, −0.22, *N* = 427, *p* = 0.0009; *I* ^2^ 61.28%; Figure 4).

Of the low‐quality studies (*k* = 23) that reported data for tests of perceptual motor function, four reported a group level impairment between post‐COVID patients and either controls or normalized data whilst one reported no group level impairment. Of the 18 studies that reported the percentage of patients with impairments 12 reported 1%–25% of patients were impaired, six reported 26%–50% of patients were impaired, and zero reported >50% of patients were impaired. The secondary domains of fluency and word finding/lexical access were the most frequently probed secondary domains. When different secondary domains were probed, no specific pattern of impairment was apparent.

### Complex attention

**FIGURE 5:**
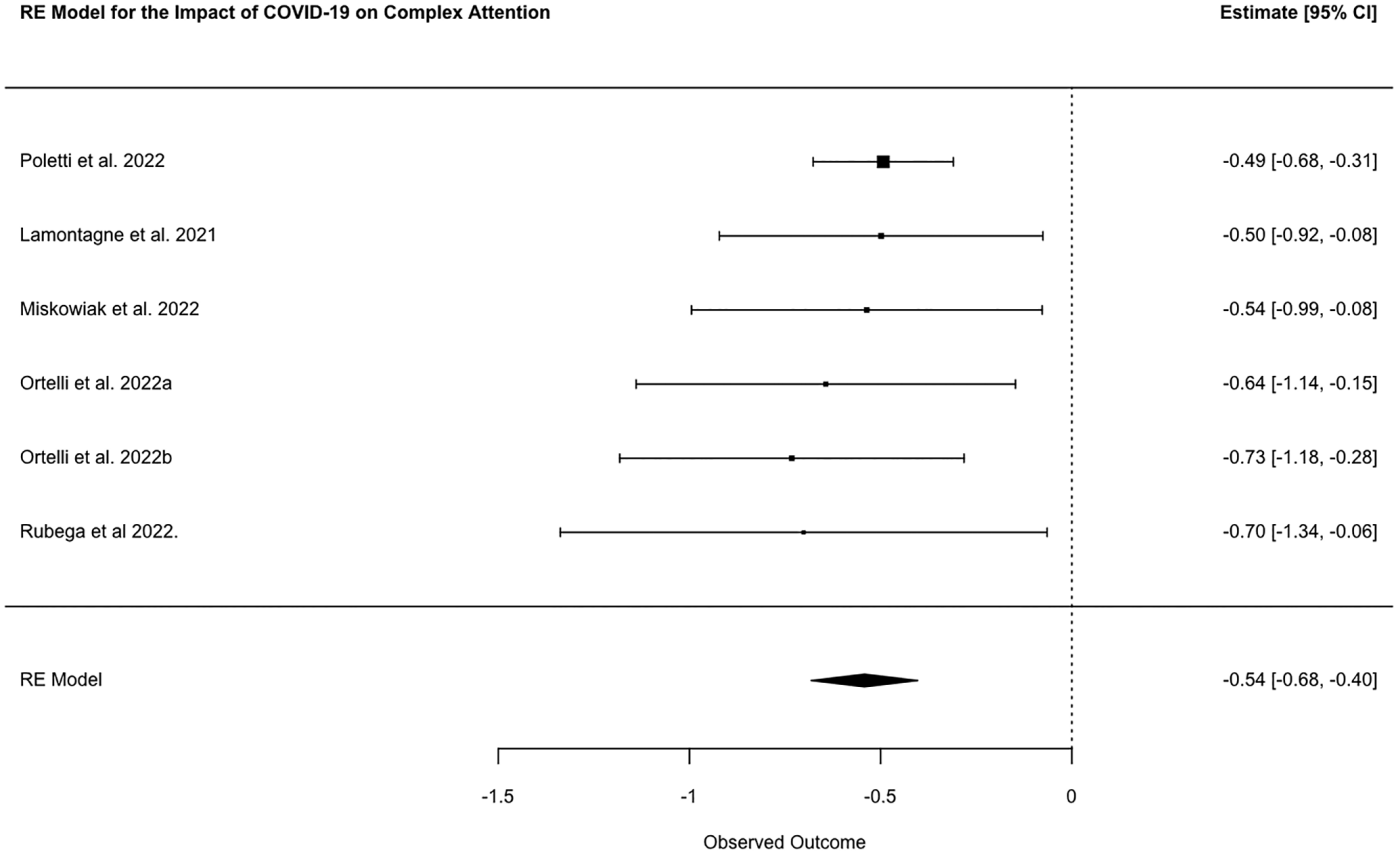
Forest plot of meta‐analysed SMDs for complex attention test scores of patients post‐COVID‐19 disease and healthy controls.

Complex attention assessments were reported by 32 studies. Symbol Digit Modalities (from Brief Repeatable Battery of Neuropsychological Tests; *n* = 13) and Continuous Performance Test (*n* = 5) were the most frequently used tasks for complex attention. A meta‐analysis of six studies showed a combined SMD between cases and controls of −0.54 (95% CI −0.68, −0.40, *N* = 870, *p* ≤ 0.0001; *I* ^2^ 0.0%; Figure 5).

Of the low‐quality studies (*k* = 19) that reported data for tests of complex attention, three reported a group level impairment between post‐COVID patients and either controls or normalized data whilst two reported no group level impairment. Of the 14 studies that reported the percentage of patients with impairments five reported 1%–25% of patients were impaired, seven reported 26%–50% of patients were impaired, and two reported >50% of patients were impaired. One study reported in detail the severity of impairment, finding in the Test for Attentional Performance that 47.5% of patients had moderate impairment and 3% had severe impairment [54].

### Visuospatial cognition

Seven studies examined visuospatial cognition with no study meeting high‐quality criteria. Six tasks were used across the studies, with Benton Judgement of Line Orientation the most frequently used (*n* = 4). Of six low‐quality studies that reported data for tests of visuospatial function, one reported a group level impairment between post‐COVID patients with symptoms at follow‐up and normalized data but reported no impairment in those without symptoms. Of the five studies that reported the percentage of patients with impairments four reported 1%–25% of patients were impaired, one reported 26%–50% of patients were impaired, and zero reported >50% of patients were impaired. No studies assessed multiple secondary domains of visuospatial cognition.

### Social cognition

Three studies examined social cognition with no studies meeting high‐quality criteria. Two of 52 patients (3.8%) scored lower than expected compared to normative scores in the Mini‐Sea Test [63]. Impairment was found in Cognition Face Emotional Discrimination but no breakdown was given [70], and outcomes were not reported in a study utilizing the Geneva Emotion Recognition Test [104].

### Overall findings

Of the seven primary DSM‐5 cognitive domains, only five were assessed by enough high‐quality studies to facilitate meta‐analysis. The results indicated all of these domains were significantly impaired in post‐COVID patients compared to healthy controls. Medium effect sizes indicating impairment were seen across executive function (SMD −0.45), learning and memory (SMD −0.55), complex attention (SMD −0.54) and language (SMD −0.54), with perceptual motor function appearing to be impacted to a greater degree (SMD −0.70) but not crossing the threshold for large effect size (Figure S4 and Table S1). Funnel plots are presented in Figure S7A–E, showing a largely symmetrical distribution across all primary domains indicating low risk of publication bias. The narrative review of low‐quality studies finds broadly similar degrees of impairment across primary domains (Figure S2), although some domains were tested more frequently (Figure S2 and Table S4). A heatmap visualizing the findings of the low‐quality studies showed no obvious pattern of domain‐specific impairment (Figure S3). All of the low‐quality papers that reported outcomes for visuospatial cognition and social cognition found some degree of impairment in post‐COVID patients versus controls or normative scores.

### Prognostic factors

Prognostic factors and their potential effects on cognition were identified by 24 studies. Five studies examined the effects of prognostic factors on overall or non‐specific cognition and so could not be included in this sub‐analysis. Twenty studies reported specific impacts of prognostic factors on stated primary cognitive domains and their impact on domain‐specific cognitive outcomes as well as quality of evidence ratings. Of the four high‐quality studies that assessed prognostic factors, a diversity of exposures and outcomes was reported. Two studies reported worse respiratory symptoms at point of admission as being significantly associated with poorer performance on complex attention tasks (*p* = 0.03) and executive function tasks (*p* = 0.02). A single study analysed the impact of baseline D‐dimer on learning and memory and found it was significantly associated with poorer performance (*p* = 0.03). Two studies examined the associations between domain‐specific cognitive impairment and admission to ICU. Both reported no significant association for performance on executive function and learning and function tasks; one of the studies also reported perceptual motor function and complex attention performance which were similarly not significantly associated with ICU admission. The prognostic factor analyses of low‐quality studies are presented alongside high‐quality studies using GRADE criteria in Table S3[32].

### Time‐point analysis

Nine studies examined primary domains at two or more time points, although three did not report outcomes (Table 1). The three high‐quality studies all showed no change in executive function or learning and memory at follow‐up. In perceptual motor function, one study showed worsening impairment and another showed no change; only one study analysed language and no change was reported. Of the two studies that reported complex attention one indicated improvement and the other no change. No high‐quality studies examined visuospatial cognition or social cognition in a longitudinal manner. The longitudinal findings of the remaining three low‐quality studies are outlined in Table 1.

**TABLE 1:** Longitudinal analysis—studies that examined primary domains in the same patients at more than one time point.

Study	Time points	Executive function	Learning and memory	Perceptual motor function	Language	Visuospatial cognition	Complex attention	Social cognition
High quality								
Andrei Appelt et al. (2022) [49] *n* = 53	0–3, 3–6 and 6–12 months	No change Residual impairment	No change Residual impairment	Worsened (between 0–3 months and 6–12 months)	Not tested	Not tested	Not tested	Not tested
Miskowiak et al. (2022) [87] *n* = 25	3 and 12 months	No change Residual impairment	No change Residual impairment	Not tested	No change Residual impairment	Not tested	No change Residual impairment	Not tested
Poletti et al. (2022) [94] *n* = 642	1, 3 and 6 months	No change Residual impairment	No change Residual impairment	No change Residual impairment	Not tested	Not tested	Improvement	Not tested
Low quality								
Ferrucci et al. (2022) [66] *n* = 76	5 and 12 months	Not tested	Improvement	Not tested	No change Residual impairment	Not tested	Improvement	Not tested
Cecchetti et al. (2022) [56] *n* = 49	2 and 10 months	No change Residual impairment	Improvement	No change Residual impairment	No change Residual impairment	No change Residual impairment	Improvement	Not tested
Shanley et al. (2022) [98] *n* = 56	Baseline after infection and 6 months	No change Residual impairment	No change Residual impairment	No change Residual impairment	No change Residual impairment	Not tested	No change Residual impairment	Not tested

*Note*: All studies that reported conducted cognitive tests at at least two time points in the same patient cohorts are included. Grey indicates the studies that either only reported outcomes at one time point (so no comparison could be made) or did not report outcomes at all. Green indicates that primary domain outcomes improved between time points, reported as significant by the authors. Yellow indicates that no significant change in primary domain outcome was found by the authors. Red indicates that primary domain outcomes worsened between time points, reported as significant by the authors.

## DISCUSSION

This systematic review and meta‐analysis describes the pattern of cognitive impairment ranging from 1 to 12 months after COVID‐19 illness, across domains, and whether there are predictors of this impairment. From 66 studies with a total of 134 different cognitive tests, evidence was found of a global impairment in cognition across the spectrum of COVID‐19 disease severity. A meta‐analysis of 10 high‐quality studies suggested moderate impairment across primary cognitive domain tests in patients post‐COVID‐19 versus controls. A greater degree of impairment was evident in perceptual motor function although this did not cross the threshold for large effect size.

Within the narrative synthesis of the remaining studies executive function was the most frequently tested domain, and a degree of impairment was reported in 69%. Similar frequencies of observed impairment were reported in learning and memory (82%), perceptual motor function (68%), language (73%) and complex attention (69%). There was a dearth of studies that evaluated visuospatial and social cognition but impairment was noted in all studies that reported outcomes in these domains. When secondary domains were examined, no pattern emerged; when a domain was impaired, it was typically impaired across its defined secondary domains.

This contrasts with the picture emerging of patients with COVID‐19 in the acute phase showing a preponderance for executive function deficits [9, 11, 12]. Neurotropic infections are thought to cause domain‐specific impairment due to the location and mechanism of their pathogenesis [34]. However, for viruses that may have a para‐infectious effect on the brain, it is less clear that specific patterns exist. Several mechanisms have been proposed to explain the nervous system manifestations of COVID‐19, including neuroinflammation, thrombotic events, cerebral endotheliopathy and autoimmune reaction, with less evidence implicating direct viral neurotropism [35, 36] and no consistent anatomical localization identified [37]. This current review adds to the literature supporting global cognitive impairment in the post‐acute phase after COVID‐19 illness, suggesting heterogeneous aetiology or the lack of a preferential anatomical location across the population of affected individuals.

This global cognitive impairment may persist over time, with little evidence from individual studies of better cognitive performance in patients evaluated 3 months post‐discharge compared to those assessed sooner [10, 38, 39]. Furthermore, impairments in executive function and processing speed have been detected 6 months after hospital discharge, and impaired Montreal Cognitive Assessment performance has been detected up to 12 months after hospital discharge, with no evidence of recovery over this time period [8, 40]. In this systematic review, a sub‐analysis of studies with more than one time point revealed longitudinal improvement in the domains of complex attention and learning and memory, most commonly at time points 10–12 months after the initial COVID‐19 illness (notably further from COVID‐19 illness than the 1‐, 3‐ and 6‐month time points more common to studies in the sub‐analysis) [48, 55, 64, 86, 93, 97]. In contrast, the only study that found any worsening of domain‐specific outcomes did so in perceptual motor function [48]. All other studies with multiple time points found no change in domain‐specific outcomes. Interestingly, one study found improvement between the 3‐ and 6‐month time points in verbal episodic memory but not in non‐verbal episodic memory, suggesting a possible secondary‐domain‐specific differential not captured elsewhere in this review [64]. It is possible then that impairment in executive function, language and perceptual motor function may persist beyond 6 months. These findings should be tempered by the fact that few studies reported longitudinal outcomes, and only three were classified as high quality by this review.

This review found that respiratory symptoms at point of admission have the best evidence of correlating with domain‐specific outcomes, especially in executive function and complex attention domains. Interestingly, in the analysis of high‐quality papers there was no indication of significant associations between ICU admission and poorer domain‐specific performance. Other potential prognostic markers identified were male sex, age, inflammatory biomarkers and mental health symptoms at time of assessment. The evidence, as assessed by GRADE, was less certain for these factors. Inflammatory biomarkers and mental health factors measured were varied and inconsistent, limiting interpretation. Additionally, several of the low‐quality studies included patients who were referred to specialist clinics with persistent symptoms or difficulties, probably over‐representing populations in which cognitive dysfunction and overlying neuropsychiatric symptoms are more common; it would be useful for future studies with broad patient inclusion criteria to investigate COVID‐19's possible interactions with neuropsychiatric conditions and delirium [12, 42]. This might also clarify which patients, if any, are at greater risk of longer term cognitive symptoms and possible cognitive decline [43], which would be highly relevant for research and public health.

The current literature displays multiple areas of weakness including small sample sizes, lack of control groups, heterogeneity of cognitive assessment tools employed and inconsistencies in reporting of cognitive test results. To mitigate these issues a meta‐analysis was performed for only high‐quality studies with larger sample sizes, healthy control groups and mitigation of confounding, with priority given to cognitive tests that most accurately tested the ascribed cognitive domain. Despite this heterogeneity remained evident in the meta‐analysis due to grouping of different acute infection severities, different tests used and varying follow‐up time points. However, given the variety of cognitive tasks used, significance testing between matched groups might also have better accounted for testing variability and varying construct validity [41].

Only a few high‐quality studies looked at domain‐specific cognitive outcomes at multiple time points, or at the impacts of prognostic factors on domain‐specific cognition. Those that did used varying follow‐up time points and varying measured exposures, making meta‐analysis of these aspects unfeasible. To elucidate any associated prognostic factors adequately powered and protocol‐supported prospective studies are required, conducted in line with the Prognostic Research Strategy Guidelines (PROGRESS) [44]. Furthermore, this review revealed a dearth of studies assessing social cognition, despite some evidence that it is as diagnostically relevant as other DSM‐5 domains in other contexts such as mild cognitive impairment [45]. The majority of evidence was drawn from high‐income countries and a lack of reporting of ethnicity makes it difficult to assess whether traditionally under‐represented groups have been adequately evaluated in this domain, affecting the generalizability of the results; a focus on traditionally neglected groups will aid in ascertaining if specific risk factors might apply to certain individuals.

Despite these limitations, this review has several scientific and clinical implications. Our meta‐analysis shows moderate impairment across domains after COVID‐19 illness, in contrast to domain‐specific impairment seen after neurotropic infections such as herpes simplex virus [34]. A standardized and systematic approach to cognitive testing following COVID‐19 infection is required, including all core DSM‐5 cognitive domains, to inform future rehabilitation approaches. Future research should focus on longitudinal evaluation of patients and include matched comparison groups to assess for causality between COVID‐19 infection and cognitive impairment. Additionally, confounding factors such as age, education level, depression/anxiety and ability to attend to a task should be considered when assessing cognition. A comparison across the spectrum of COVID‐19 disease and inclusion of individuals with comorbid neuropsychiatric illness is required, which will ultimately lead to better elucidation of how COVID‐19 affects domain‐specific cognitive outcomes in the medium and long term.

## AUTHOR CONTRIBUTIONS

**Jack B. Fanshawe:** Conceptualization; formal analysis; writing – original draft; data curation; methodology. **Brendan F. Sargent:** Formal analysis; writing – original draft; data curation; methodology; visualization. **James B. Badenoch:** Investigation; writing – review and editing; data curation; methodology. **Aman Saini:** Investigation; writing – review and editing. **Cameron J. Watson:** Formal analysis; writing – review and editing; methodology; visualization. **Aleksandra Pokrovskaya:** Investigation; writing – review and editing. **Daruj Aniwattanapong:** Investigation; writing – review and editing. **Isabella Conti:** Investigation; writing – review and editing. **Charles Nye:** Investigation; writing – review and editing. **Ella Burchill:** Investigation; writing – review and editing. **Zain U. Hussain:** Investigation; writing – review and editing. **Khanafi Said:** Investigation; writing – review and editing. **Elinda Kuhoga:** Investigation; writing – review and editing. **Kukatharmini Tharmaratnam:** Investigation; formal analysis; visualization; writing – review and editing. **Sophie Pendered:** Project administration; visualization; writing – review and editing. **Bernard Mbwele:** Conceptualization; writing – review and editing; supervision; methodology. **Maxime Taquet:** Writing – review and editing; visualization. **Greta K. Wood:** Writing – review and editing; conceptualization; supervision. **Jonathan P. Rogers:** Writing – review and editing; supervision. **Adam Hampshire:** Conceptualization; methodology; supervision; writing – review and editing. **Alan Carson:** Conceptualization; methodology; writing – review and editing; supervision. **Anthony S. David:** Conceptualization; methodology; writing – review and editing; supervision. **Benedict D. Michael:** Writing – review and editing; methodology; supervision; conceptualization. **Timothy R. Nicholson:** Conceptualization; methodology; writing – review and editing; supervision. **Stella‐Maria Paddick:** Conceptualization; methodology; writing – review and editing; supervision. **Charles E. Leek:** Conceptualization; methodology; supervision; writing – review and editing.

## CONFLICT OF INTEREST STATEMENT

BDM declares payments for the UMASS lecture 2023 and for medicolegal work unrelated to the topic of this manuscript or the manuscript itself. BDM is also the Encephalitis Society Vice Chair for which he receives no remuneration. AC has received a CSO Scotland grant for cognitive phenotyping of post‐covid symptoms for work unrelated to this manuscript. AC has been a Virginia University Grand Rounds speaker honorarium on functional cognitive disorders and has received payment for expert testimony on a range of neuropsychiatric topics unrelated to this manuscript. AC is also a paid editor for the Journal of Neurology, Neurosurgery and Psychiatry and a president of FND society for which he receives no remuneration. S‐MP reports receiving travel reimbursement for acting as an invited speaker at two conferences; (1) International Brain Research Organisation—Regional African Neurology Training (2023) and (2) Alzheimer's Association—Dementia in LMICs (2022). S‐MP also sits on the executive committee of the RCPsych VIPSIG, an unpaid role. KS has received support from the Tanzanian Medical Students Association for support to attend academic conferences unrelated to this work. ASD is supported by the NIHR Biomedical Research Centre, University College Hospital London and is the president of the International Neuropsychiatric Association. JPR acknowledges a grant from the Wellcome trust paid to his institution to cover salary and research expenses for work unrelated to this manuscript. MT acknowledges grants from the MQ Mental Health Research and Wolfson Foundation to investigate mechanisms of post‐COVID cognitive deficits. AH is co‐founder and director of H2CD Inc, a software company that provides online assessment technology for research and healthcare purposes unrelated to this manuscript. GKW, KT, BFS, CN, JBF, DA, BM, SP, AP, ZUH, EB, AS, ECL, EK, TRN, JBB, IC have no COI to declare.

## Supporting information


Data S1:

## Data Availability

The data that support the findings of this study are available on reasonable request from the corresponding author. These data were derived from the studies referenced in this manuscript.
