# Correction to “Cognitive Domains Affected Post‐COVID‐19; a Systematic Review and Meta‐Analysis”

**DOI:** 10.1111/ene.70253

**Published:** 2025-06-20

J. B. Fanshawe, B. F. Sargent, J. B. Badenoch, et al., “Cognitive Domains Affected Post‐COVID‐19; a Systematic Review and Meta‐Analysis,” European Journal of Neurology 32 (2025):e16181. https://doi.org/10.1111/ene.16181.

An author's name and affiliation were incorrectly published as “Charles E. Leek, Department of Psychology, University of Liverpool, Liverpool, UK.” It should have been “E. Charles Leek, School of Psychology, University of Southampton, Southampton, UK.”

We apologize for this error.

