# Supplementary material for: Cognitive domains affected post‐COVID‐19; a systematic review and meta‐analysis
Source: Eur J Neurol. 2024 Feb 20;32(1):e16181. doi: 10.1111/ene.16181 (PMC11618111; doi:10.1111/ene.16181)
Supplement: Supplementary file 1 — Data S1: [file ENE-32-e16181-s001.pdf]

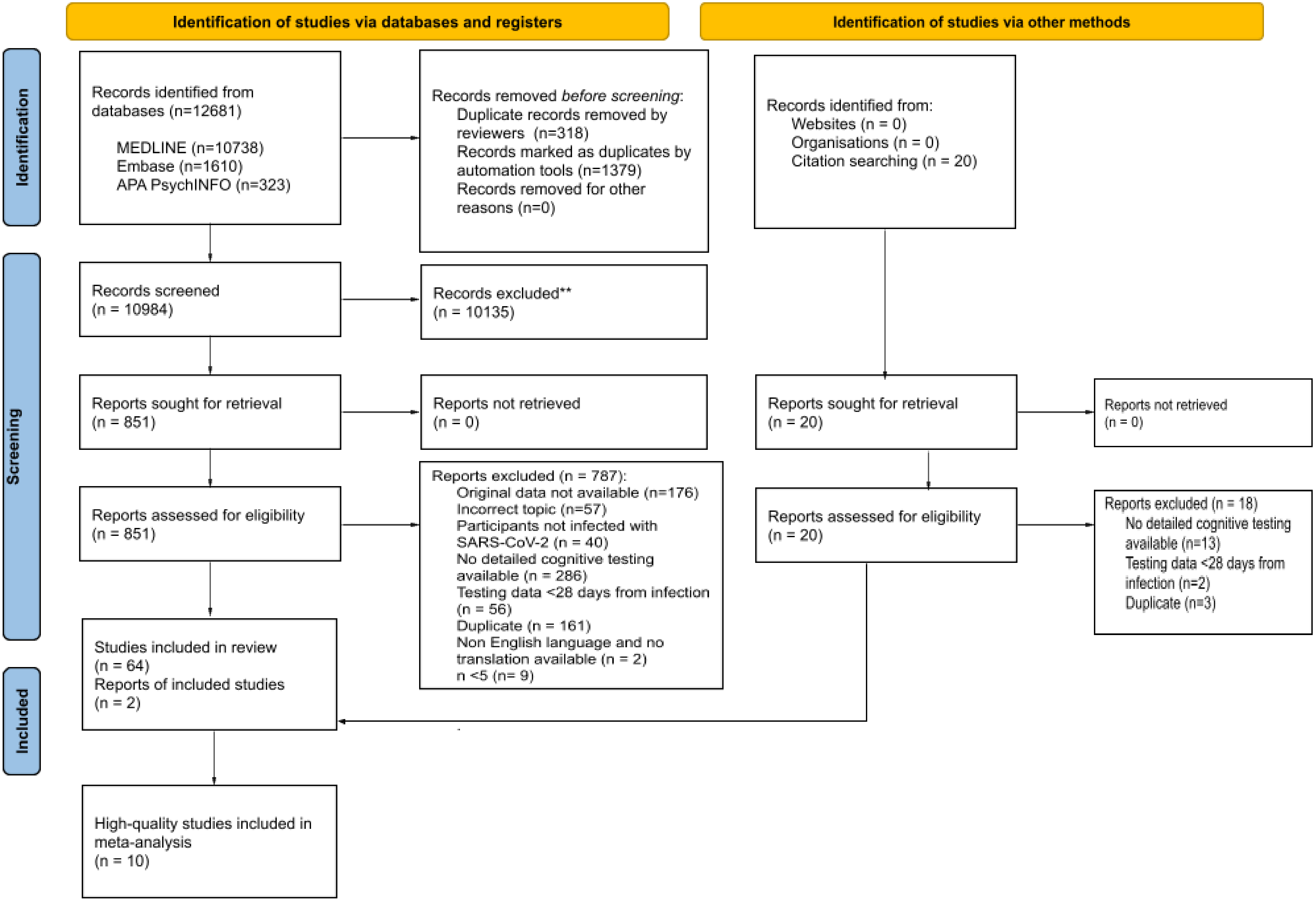

Identification of studies via databases and registers
Identification of studies via other methods
Identification
Records identified from databases (n=12681)
MEDLINE (n=10738)
Embase (n=1610)
APA PsychINFO (n=323)
Records removed *before screening*:
Duplicate records removed by reviewers (n=318)
Records marked as duplicates by automation tools (n=1379)
Records removed for other reasons (n=0)
Records identified from:
Websites (n = 0)
Organisations (n = 0)
Citation searching (n = 20)
Screening
Records screened (n = 10984)
Records excluded** (n = 10135)
Reports sought for retrieval (n = 851)
Reports not retrieved (n = 0)
Reports sought for retrieval (n = 20)
Reports not retrieved (n = 0)
Reports assessed for eligibility (n = 851)
Reports excluded (n = 787):
Original data not available (n=176)
Incorrect topic (n=57)
Participants not infected with SARS-CoV-2 (n = 40)
No detailed cognitive testing available (n = 286)
Testing data <28 days from infection (n = 56)
Duplicate (n = 161)
Non English language and no translation available (n = 2)
n <5 (n= 9)
Reports assessed for eligibility (n = 20)
Reports excluded (n = 18)
No detailed cognitive testing available (n=13)
Testing data <28 days from infection (n=2)
Duplicate (n=3)
Included
Studies included in review (n = 64)
Reports of included studies (n = 2)
High-quality studies included in meta-analysis (n = 10)

Supplementary Figure 1 - PRISMA diagram of study selection process

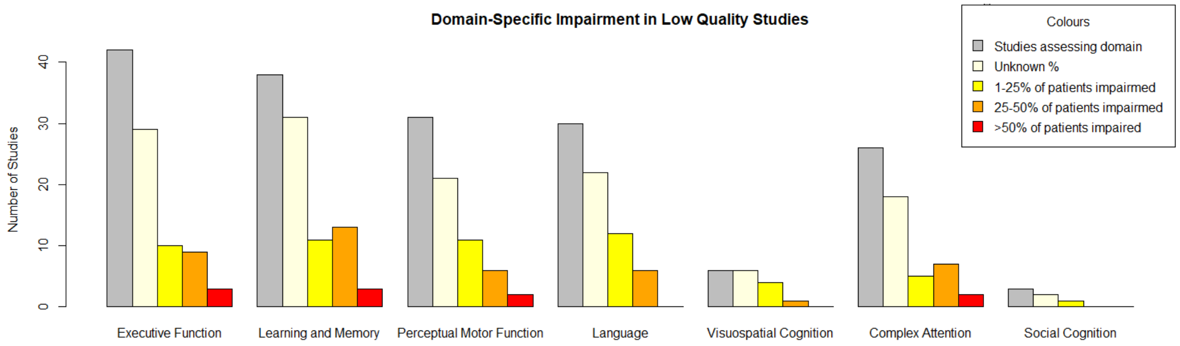

Supplementary Figure 2: Number of studies that assessed given domains finding impairment

Multibox plots of the number of studies assessing a given domain (grey) and the numbers of those studies reporting different proportions of their patient populations affected. Pale yellow represents the number of studies that reported impairment in their population but did not specify proportion, and the remaining colours indicate the proportion of the patient population reported as affected. This is trichotomised into 1-25% affected (yellow), 25-50% affected (orange) and over 50% affected (red).

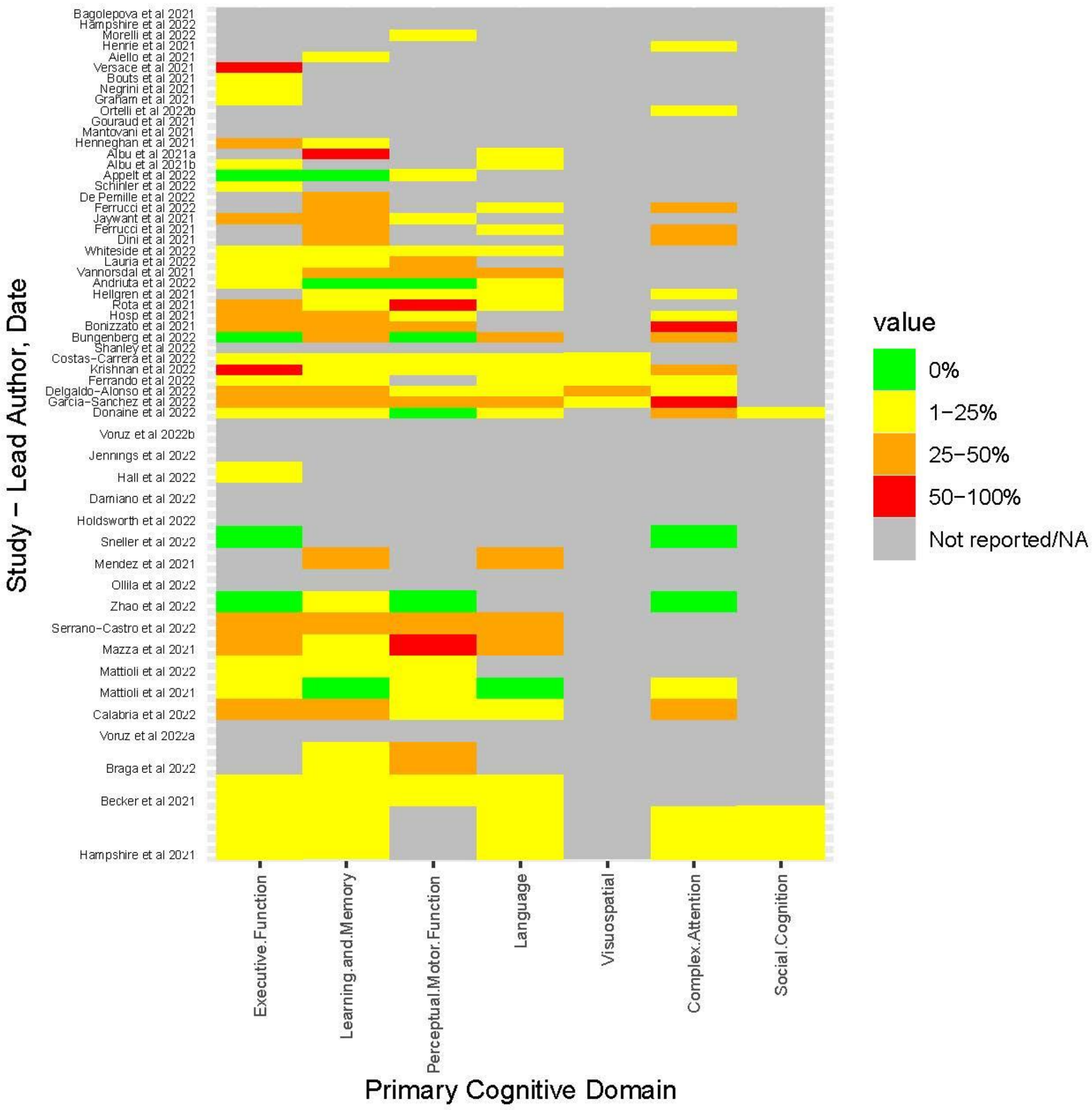
Bagolepova et al 2021
Hampshire et al 2022
Morelli et al 2022
Henrie et al 2021
Aiello et al 2021
Versace et al 2021
Bouts et al 2021
Negrini et al 2021
Graham et al 2021
Ortelli et al 2022b
Gouraud et al 2021
Mantovani et al 2021
Henneghan et al 2021
Albu et al 2021a
Albu et al 2021b
Appelt et al 2022
Schinler et al 2022
De Pemille et al 2022
Ferrucci et al 2022
Jaywant et al 2021
Ferrucci et al 2021
Dini et al 2021
Whiteside et al 2022
Lauria et al 2022
Vannorsdal et al 2021
Andriuta et al 2022
Hellgren et al 2021
Rota et al 2021
Hosp et al 2021
Bonizzato et al 2021
Bungenberg et al 2022
Shanley et al 2022
Costas−Carrera et al 2022
Krishnan et al 2022
Ferrando et al 2022
Delgado−Alonso et al 2022
Garcia−Sanchez et al 2022
Donaine et al 2022
Voruz et al 2022b
Jennings et al 2022
Hall et al 2022
Damiano et al 2022
Holdsworth et al 2022
Sneller et al 2022
Mendez et al 2021
Ollila et al 2022
Zhao et al 2022
Serrano−Castro et al 2022
Mazza et al 2021
Mattioli et al 2022
Mattioli et al 2021
Calabria et al 2022
Voruz et al 2022a
Braga et al 2022
Becker et al 2021
Hampshire et al 2021
Study − Lead Author, Date
Executive.Function
Learning.and.Memory
Perceptual.Motor.Function
Language
Visuospatial
Complex.Attention
Social.Cognition
Primary Cognitive Domain
value
0%
1−25%
25−50%
50−100%
Not reported/NA

Supplementary Figure 3: Heatmap of impairment per domain across all low quality studies, weighted by cohort size.

Studies are ordered and weighted by population size. 1 row = 5-100 patients , 2 rows = 101-500 patients, 3 rows = >501 patients.

Grey colour represents a study that either did not test a primary domain at all, or if it was tested the outcome was not reported at all. Studies that report percentage of patients impaired are categorised as 0% of patients impaired, 1-25% of patients impaired, 26-50% of patients impaired and >50% of patients impaired, as per Table 1. If a study reported impairment but did not report detailed outcomes, such that percentage of patents affected could not be inferred or calculated, the colour yellow has been used to represent impairment (of unknown degree).

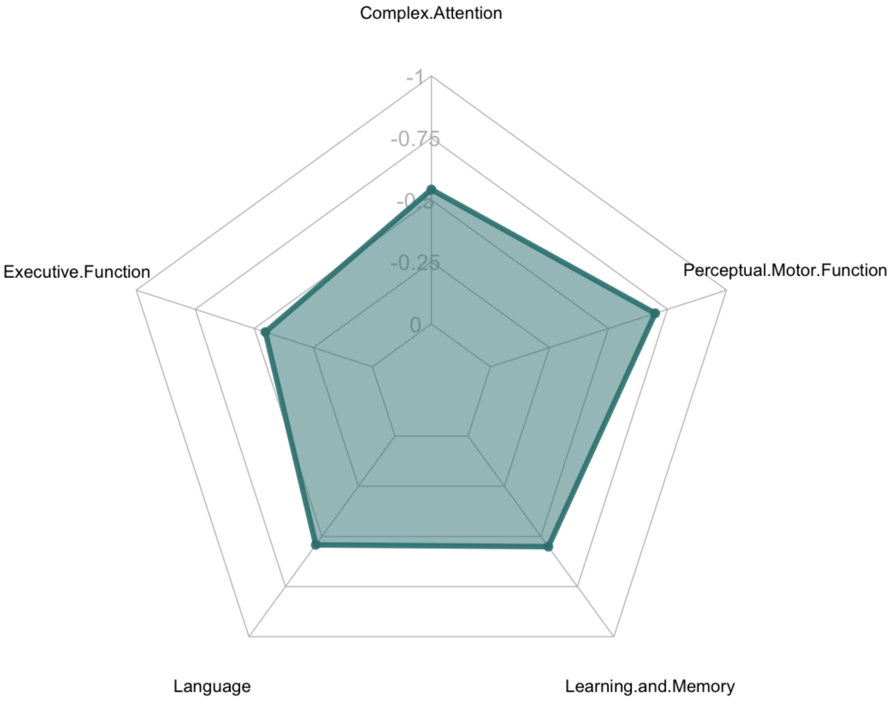

Supplementary Figure 4: Radar plot of meta-analysed SMDs for primary cognitive domains of patients post COVID-19 disease and healthy controls

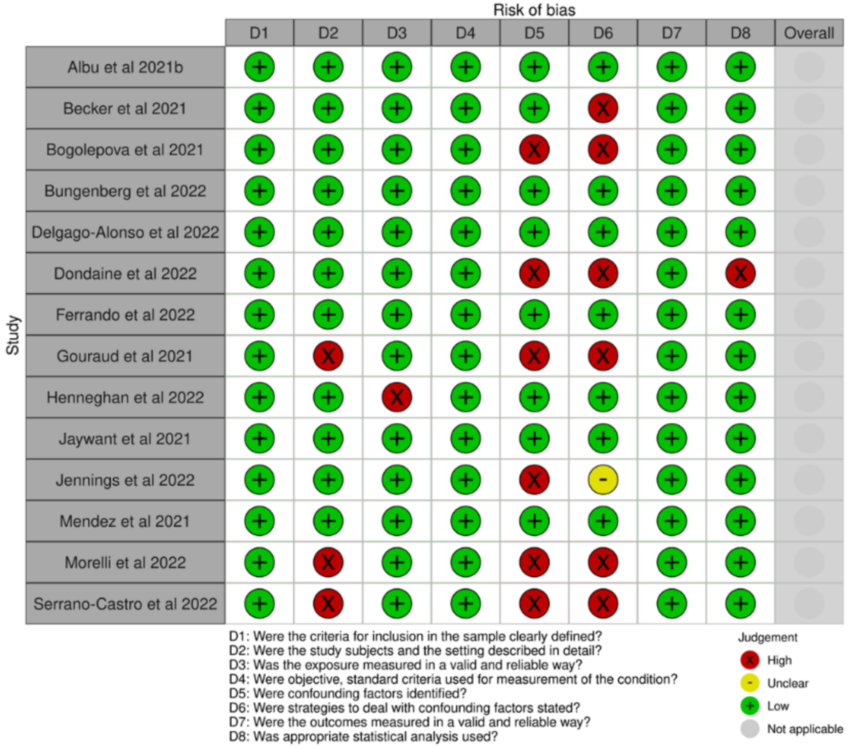
Risk of bias
D1
D2
D3
D4
D5
D6
D7
D8
Overall
Study
Albu et al 2021b
Becker et al 2021
Bogolepova et al 2021
Bungenberg et al 2022
Delgago-Alonso et al 2022
Dondaine et al 2022
Ferrando et al 2022
Gouraud et al 2021
Henneghan et al 2022
Jaywant et al 2021
Jennings et al 2022
Mendez et al 2021
Morelli et al 2022
Serrano-Castro et al 2022
D1: Were the criteria for inclusion in the sample clearly defined?
D2: Were the study subjects and the setting described in detail?
D3: Was the exposure measured in a valid and reliable way?
D4: Were objective, standard criteria used for measurement of the condition?
D5: Were confounding factors identified?
D6: Were strategies to deal with confounding factors stated?
D7: Were the outcomes measured in a valid and reliable way?
D8: Was appropriate statistical analysis used?
Judgement
High
Unclear
Low
Not applicable

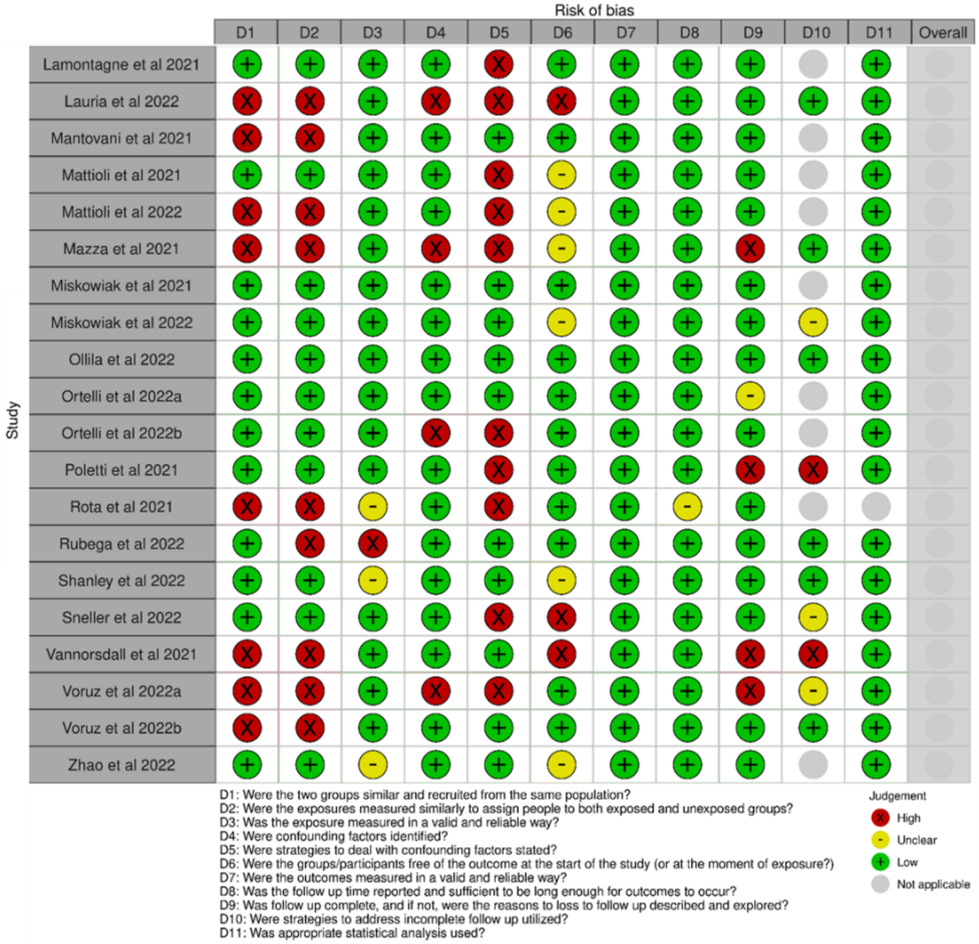
Risk of bias
D1
D2
D3
D4
D5
D6
D7
D8
D9
D10
D11
Overall
Study
Lamontagne et al 2021
Lauria et al 2022
Mantovani et al 2021
Mattioli et al 2021
Mattioli et al 2022
Mazza et al 2021
Miskowiak et al 2021
Miskowiak et al 2022
Ollila et al 2022
Ortelli et al 2022a
Ortelli et al 2022b
Poletti et al 2021
Rota et al 2021
Rubega et al 2022
Shanley et al 2022
Sneller et al 2022
Vannorsdall et al 2021
Voruz et al 2022a
Voruz et al 2022b
Zhao et al 2022
D1: Were the two groups similar and recruited from the same population?
D2: Were the exposures measured similarly to assign people to both exposed and unexposed groups?
D3: Was the exposure measured in a valid and reliable way?
D4: Were confounding factors identified?
D5: Were strategies to deal with confounding factors stated?
D6: Were the groups/participants free of the outcome at the start of the study (or at the moment of exposure?)
D7: Were the outcomes measured in a valid and reliable way?
D8: Was the follow up time reported and sufficient to be long enough for outcomes to occur?
D9: Was follow up complete, and if not, were the reasons to loss to follow up described and explored?
D10: Were strategies to address incomplete follow up utilized?
D11: Was appropriate statistical analysis used?
Judgement
High
Unclear
Low
Not applicable

Study	D1	D2	D3	D4	D5	D6	D7	D8	D9	D10	D11	Overall
Aiello et al 2021	X	X	X	+	+	X	+	X	X	X	+	
Albu et al 2021a	X	X	+	+	X	+	+	+	+	+	+	
Appelt et al 2022	+	+	+	X	X	+	+	+	+	-	+	
Bonizzato et al 2021	X	X	+	+	X	+	+	+	+			
Bouts et al 2021	X	X	-	X	X	-	+	+	+			
Braga et al 2022	X	X	+	+	+	+	+	+	+	X	+	
Cecchetti et al 2022	+	+	+	+	+	+	+	+	X	-	+	
Costas-Carrera et al 2022	X	X	+	+	+	X	+	+	X	X	+	
Crivelli et al 2022	+	+	+	X	-	+	+	+	+	+	+	
Damiano et al 2022	X	X	+	+	+	+	+	+	-		+	
Dini et al 2021	X	X	-	X	X	-	+	+	+		+	
Ferrucci et al 2021	X	X	+	+	X	-	+	+	+		+	
Ferrucci et al 2022	X	X	+	X	X	-	+	+	X	X	+	
Graham et al 2021	+	+	X	+	+	+	+	+	+		+	
Hampshire et al 2021	+	+	X	+	+	-	+	X	X	X	+	
Hampshire et al 2022	+	X	-	+	+	-	+	+	X	X	+	
Hellgren et al 2021	X	X	+	+	+	-	+	+	+		+	
Hosp et al 2021	X	X	+	+	+	+	+	+	X	+	+	
Huang et al 2021	+	+	+	X	X	-	+	+	+		+	

Risk of bias; Study

D1: Were the two groups similar and recruited from the same population?
D2: Were the exposures measured similarly to assign people to both exposed and unexposed groups?
D3: Was the exposure measured in a valid and reliable way?
D4: Were confounding factors identified?
D5: Were strategies to deal with confounding factors stated?
D6: Were the groups/participants free of the outcome at the start of the study (or at the moment of exposure)?
D7: Were the outcomes measured in a valid and reliable way?
D8: Was the follow up time reported and sufficient to be long enough for outcomes to occur?
D9: Was follow up complete, and if not, were the reasons to loss to follow up described and explored?
D10: Were strategies to address incomplete follow up utilized?
D11: Was appropriate statistical analysis used?

Judgement
X High
- Unclear
+ Low
Not applicable

Supplementary Figure 5a-c: Study Risk of Bias Tables

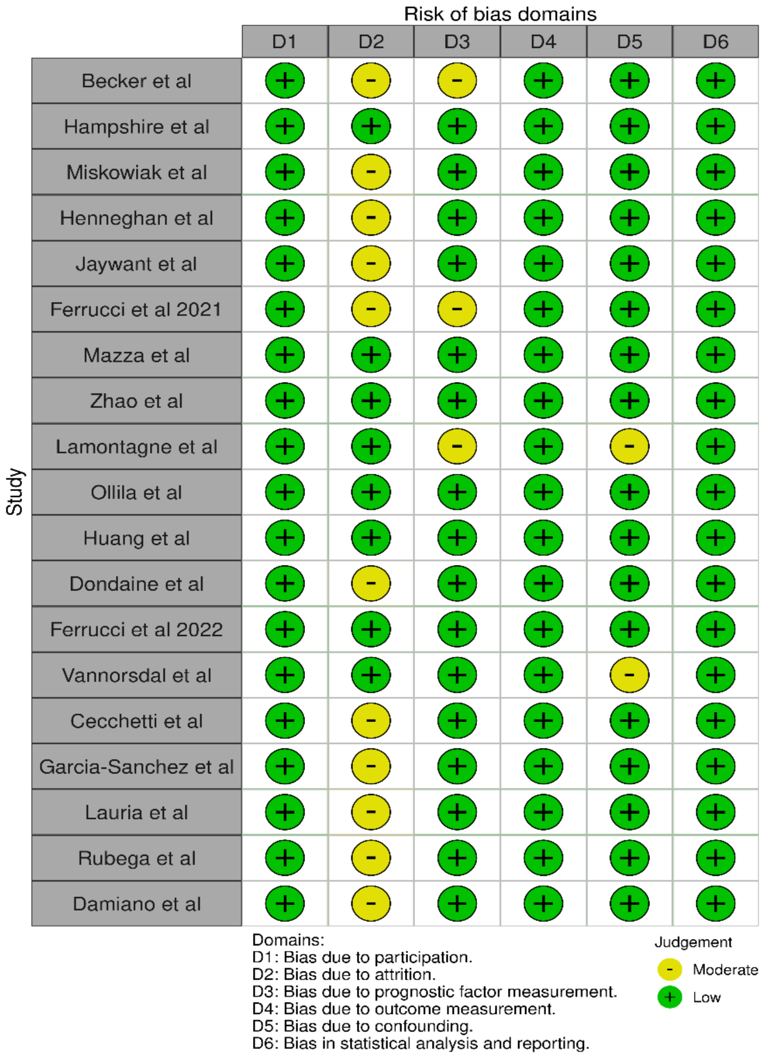

Supplementary Figure 6: Prognostic Study Risk of Bias

Funnel Plot of Studies Examining the Impact of COVID-19 on Executive Function

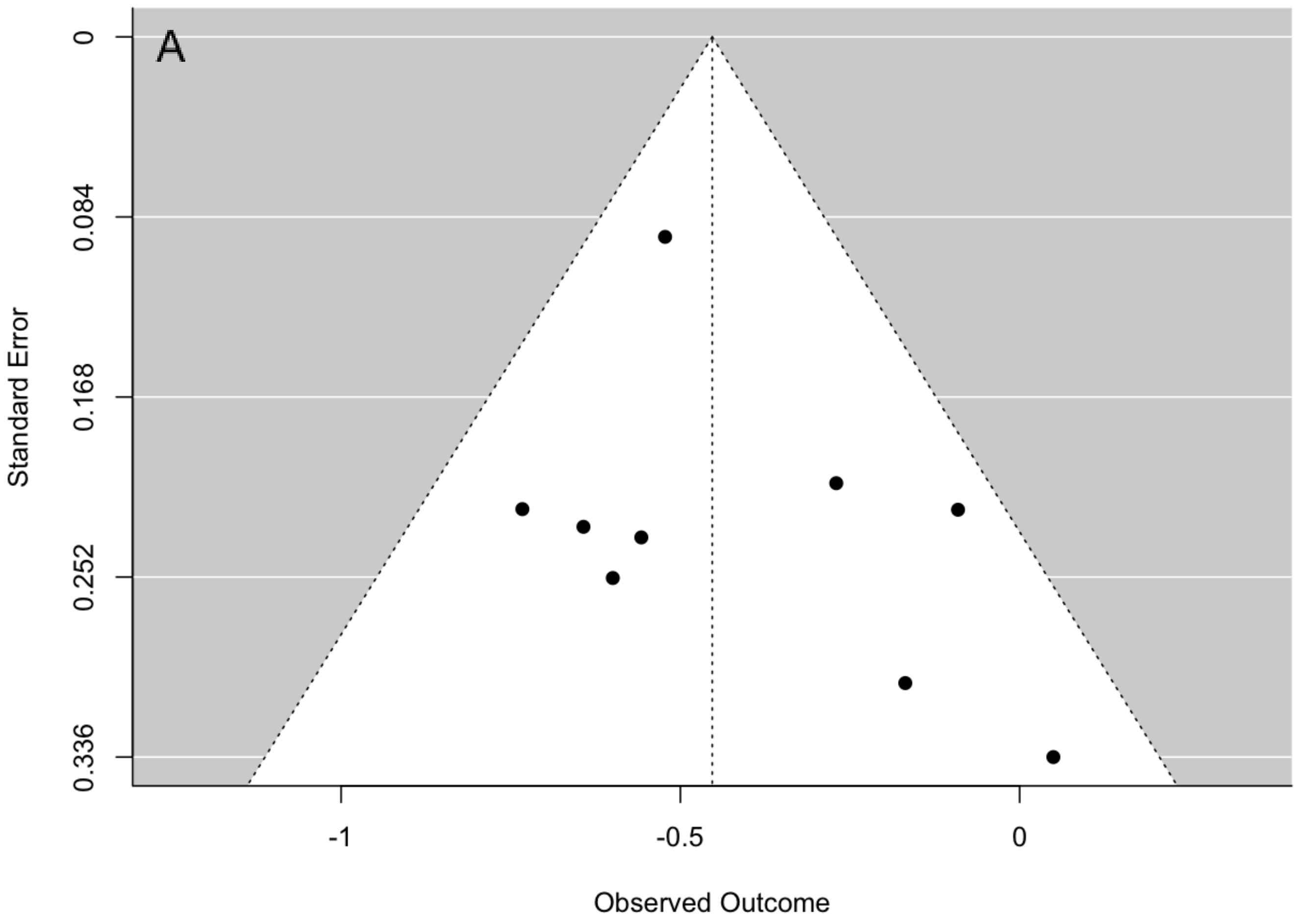

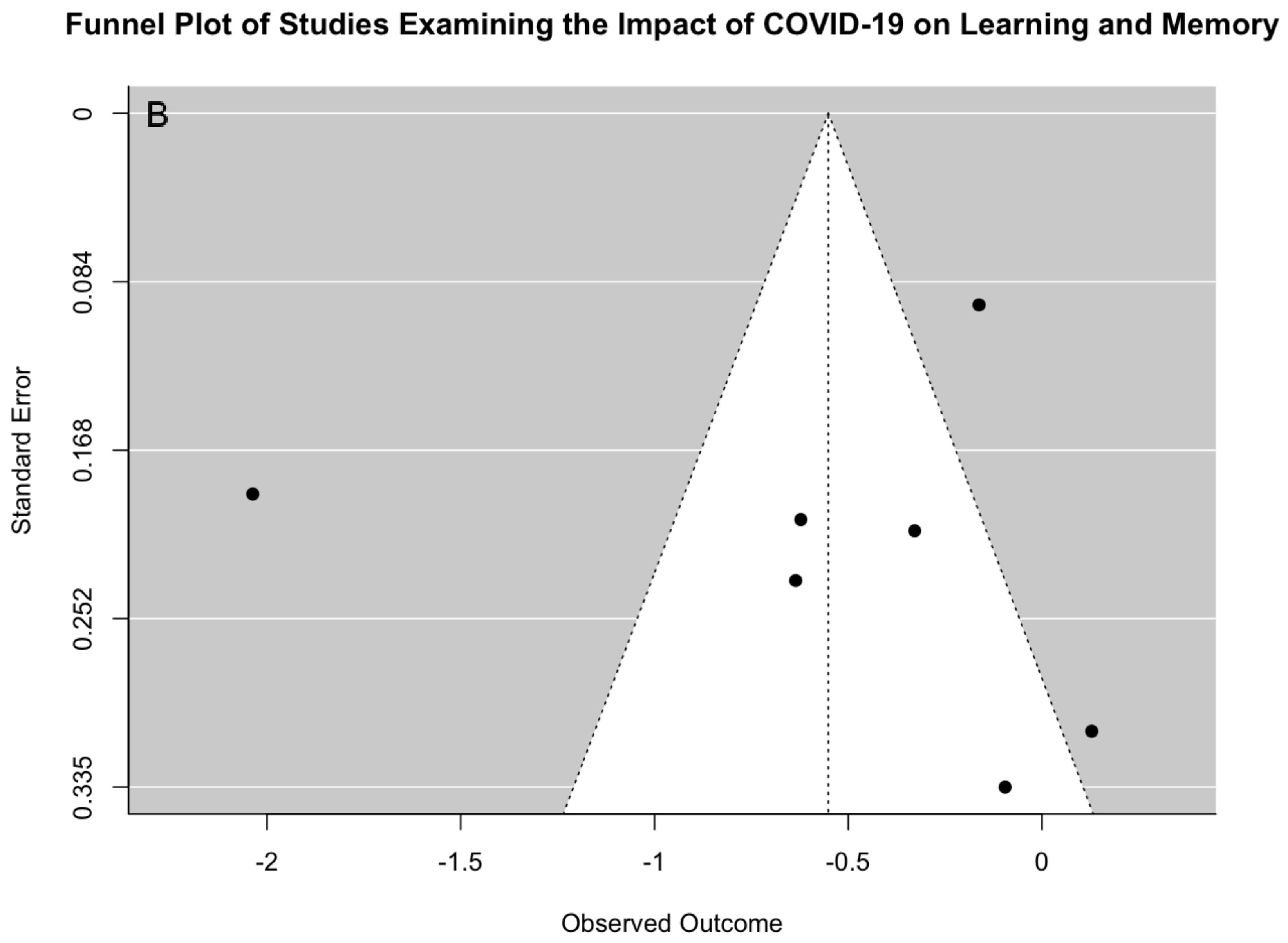
Funnel Plot of Studies Examining the Impact of COVID-19 on Learning and Memory
B
Standard Error
0
0.084
0.168
0.252
0.335
-2
-1.5
-1
-0.5
0
Observed Outcome

Funnel Plot of Studies Examining the Impact of COVID-19 on Perceptual Motor Functi

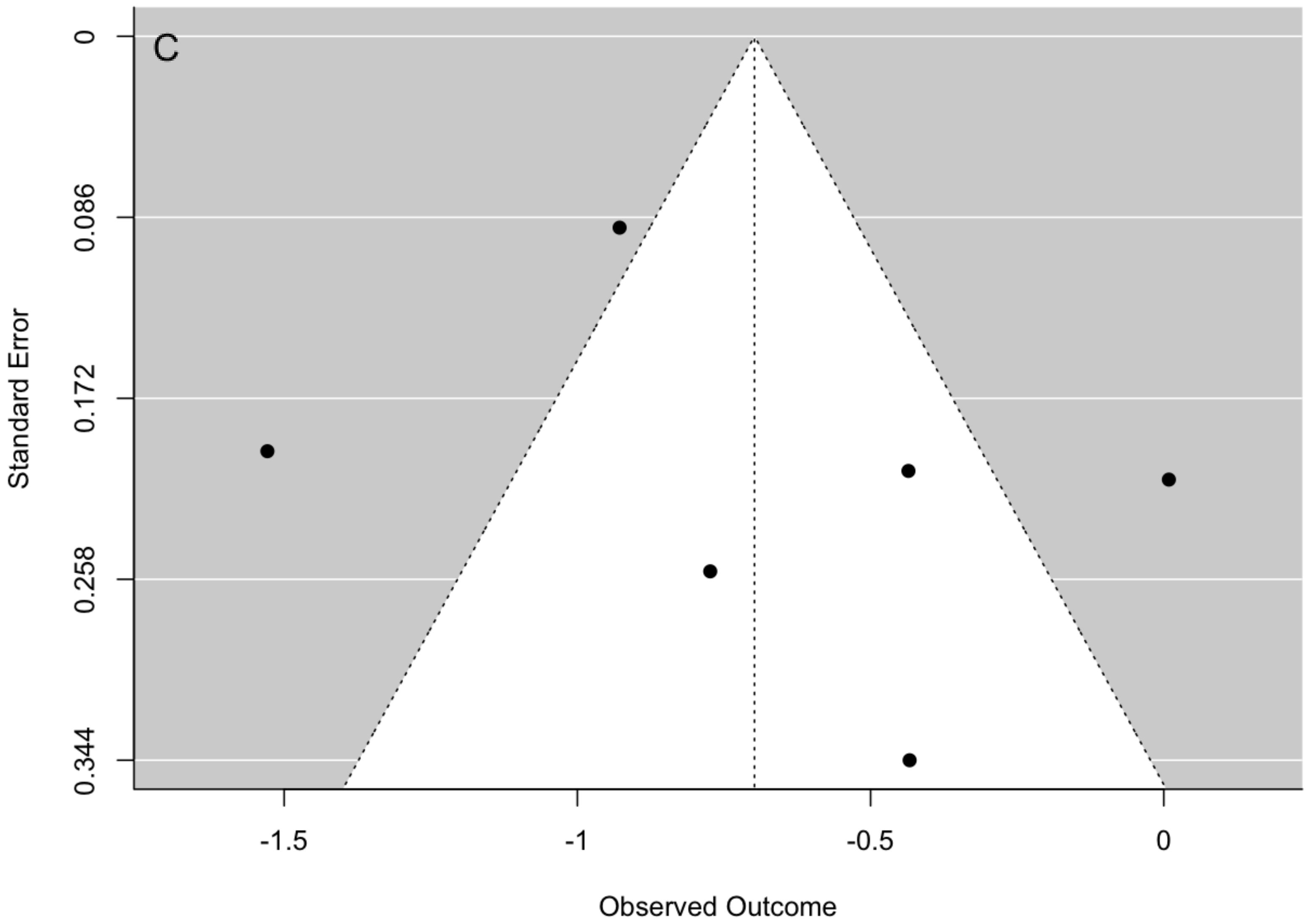

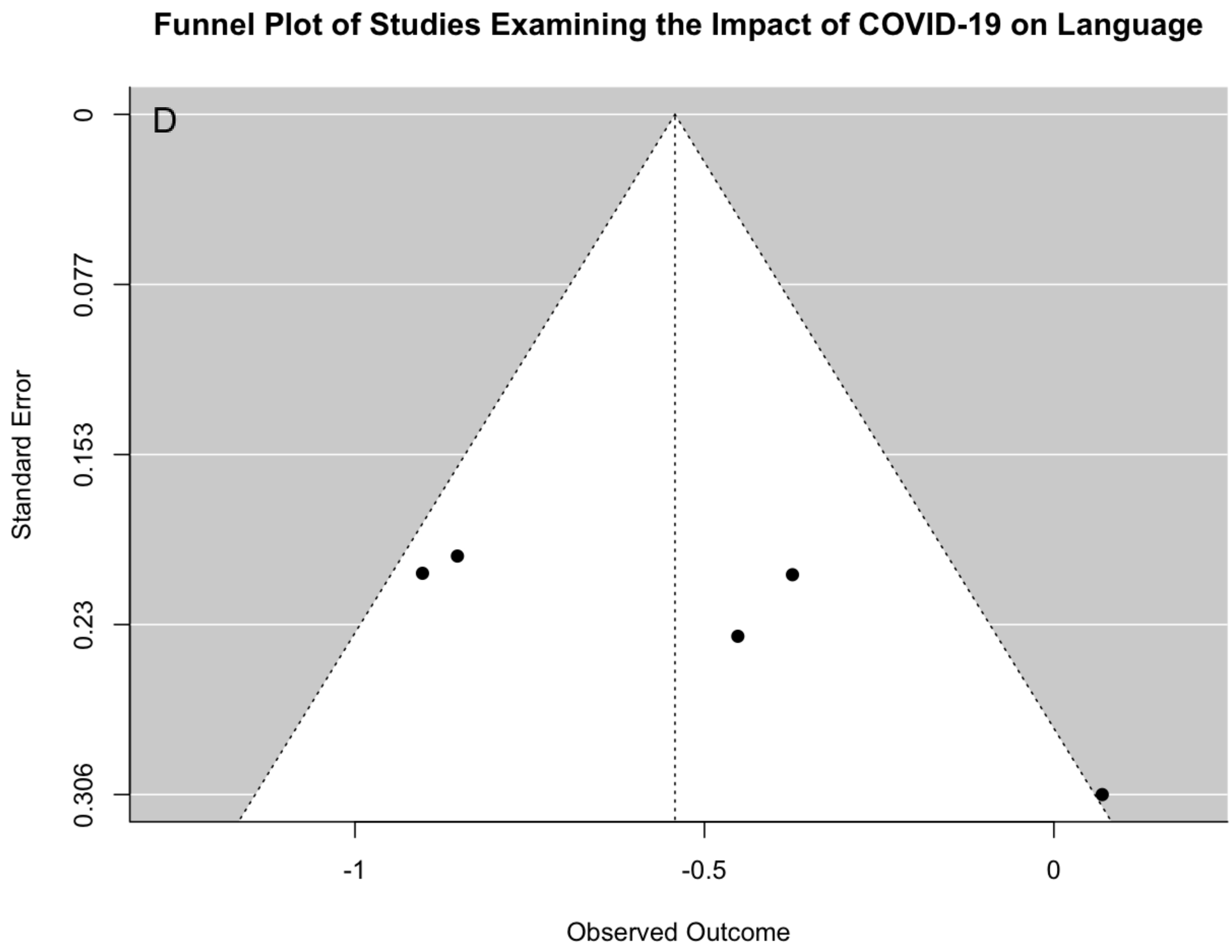

Funnel Plot of Studies Examining the Impact of COVID-19 on Language
D
Standard Error
0
0.077
0.153
0.23
0.306
-1
-0.5
0
Observed Outcome

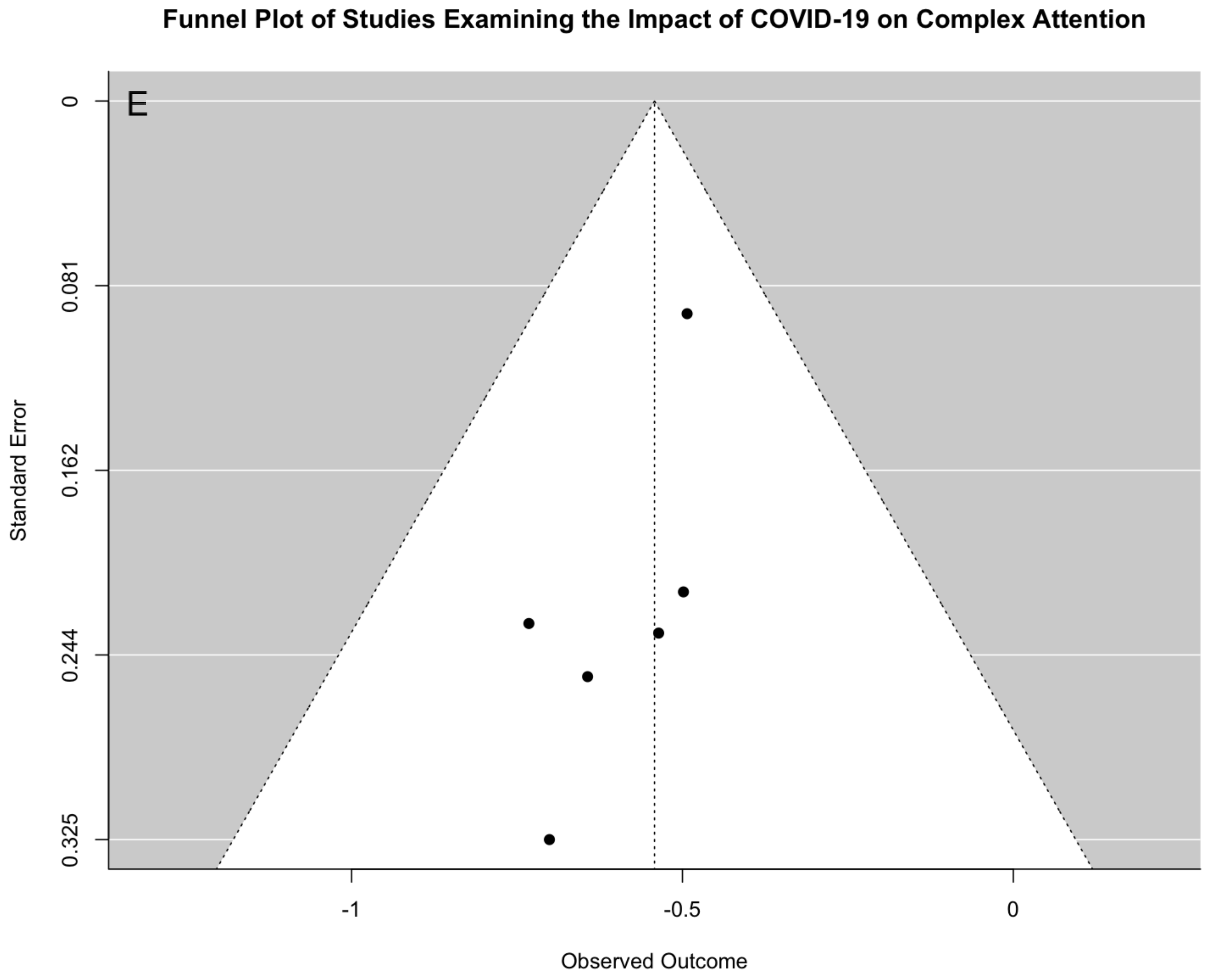

Funnel Plot of Studies Examining the Impact of COVID-19 on Complex Attention
E
Standard Error
0
0.081
0.162
0.244
0.325
-1
-0.5
0
Observed Outcome

Supplementary Figure 7A-E: Forest plots of studies examining the impact of COVID-19 illness on subsequent outcomes on tests assessing (A) executive function (B) learning and memory (C) perceptual motor function (D) language and (E) complex attention

Domain	Effect Size (SMD)	Confidence Intervals	p-value	Number of studies	Heterogeneity (%)
**Executive Function**	-0.4531	-0.5901 - -0.3160	*** <0.0001	9	9.1
**Learning and memory**	-0.5513	-1.0903 - -0.0124	* 0.045	7	92.62
**Perceptual Motor Function**	-0.6979	-1.1299 - -0.2260	** 0.0015	6	87.08
**Language**	-0.5421	-0.8610 - -0.2233	*** 0.0009	5	61.28
**Complex Attention**	-0.5422	-0.6815 - -0.4030	*** < 0.0001	6	0

Supplementary Table 1: Summary of meta-analysis of standardised mean differences between patients post COVID-19 and healthy controls * denotes p <0.05, ** denotes p< 0.01, *** p <0.001

Supplementary Table 2: outline of study characteristics

Study	Country	Single or multicentre	Setting at point of assessment	Control group used	Timing of study	Inclusion and exclusion Criteria	Number of post COVID-19 participants	Number of controls	Number of patients by location treated	Number of patients by method of COVID-19 diagnosis	Gender	Neurological complications of COVID-19 or pre-existing cognitive impairment/ neurological conditions	Ethnicity	Education level (years, mean (SD) unless stated)	Age (mean (SD) unless stated)	Time from infection – days (mean (SD) unless stated
Mattioli et al 2022	Italy	Single	Discharged/remained in community if never admitted	No control	NR	older than 18 years, who had been previously affected by symptomatic COVID-19 (confirmed diagnosis by means of a positive result on a molecular nasopharyngeal swab). They were enrolled in an observational study, aimed at prospectively evaluating the health status after COVID-19 and were all examined a mean of 4 months after diagnosis.	215	0	Hospitalised (not ITU): 76%<br>ITU (not I and V): 4%<br>ITU (I and V): 20%	Laboratory confirmed: 100%	Male: 48%<br>Female: 52%	No exclusion criteria.	NR	12 (5-18)	ITU: 60 (9.9)<br>Non-ITU: 46.9 (9.4)	120

Vannorsdall et al 2021	USA	Single	Rehabilitation facility and remained in community if never admitted	No control	July 2020 – January 2021	Acute illness from COVID-19 requiring ≥48 hours of ITU care or ongoing pulmonary and/or rehabilitation needs at the time of hospital discharge or (2) persistent symptoms at 4–6 weeks after acute infection without hospitalization. Residual symptoms prompting referral included persistent pulmonary issues, dyspnea, dysautonomia, fatigue, cognitive complaints, pain, and other nonresolving symptoms after COVID-19 infection	82	0	Community/ Hospitalised (not ITU): 42%<br>ITU (not I and V)<br>ITU (I and V)<br>ITU (I and V not stated): 58%	Laboratory confirmed: 100%	Male: 42%<br>Female: 58%	Presenting with persistent symptoms and no exclusion criteria.	White: 35%<br>Asian: 2%<br>Black: 54%<br>Hispanic: 9%	14.7 (3.1)	54.5 (14.6)	126.5 (70.1)

Mendez et al 2021	Spain	Single centre	Discharged	No control	March to April 2020	Inclusion: Diagnosed with COVID-19 via PCR, referred to the COVID-19 outpatient clinic<br><br>Exclusion: patients aged ≥ 85 or < 18 years, non-Spanish speaking subjects, nursing-home residents, pre-existing dementia/cognitive brain injury with cognitive sequelae, alcohol/substance use disorder and previous major psychiatric disorders.	179	0	Hospitalised (not ITU): 81%<br>ITU (not I and V): 6%<br>ITU (I and V): 13%	Laboratory confirmed: 100%	Male: 59%<br>Female: 41%	Patients presenting with subjective symptoms. Excluded if significant comorbidity or pre-existing cognitive impairments	NR	Median (IQR): years in education<br><br>11 (8-16)	<50: 29.1%<br>50-69: 52.5%<br>>70: 18.4%	60 (30)
Dini et al 2021	Italy	Multicentre	Discharged	No control	NR	Patients recovering from COVID-19 hospitalization who required	77	0	Hospitalised (not ITU): 58%<br>ITU (not I and V) 33%<br>ITU (I and V): 9%	Laboratory confirmed: 100%	Male: 74%<br>Female: 26%	No exclusion criteria	NR	NR	Range: 22-77	150

						different types of oxygen/ventilation therapy										
Graham et al 2021	USA	Single Centre	Discharged	SARS-CoV-2 patients with post-acute viral syndrome	May and November 2020	NR	100	0	Community: 100%	Laboratory confirmed: 50% Clinically suspected: 50%	Male: 30% Female: 70%	No significant difference between cohorts	White: 88% Black: 6% Asian 2% American Indian or Alaskan native: 1% Other: 3%	NR	43.2 (11.3)	5.27 (1.83) months
Bogolepova et al 2021	Russia	Single Centre	Community	No control	Not stated	Participants aged 22 to 71 years who have had COVID-19 5.4 months ago. Inclusion criterion: cognitive complaints, fatigue, and emotional disturbances.<br><br>No exclusion criteria	100	0	Community (not further specified):100	Laboratory confirmed: 100	Male: 59% Female: 41%	Pre-existing Cognitive impairment in 33%	NR	NR	49,71± 11,10	5.4 months

Ferrucci et al 2021	Italy	Single centre	Discharged	No controls	February to April 2020.	patients hospitalized for SARS-CoV-2 infection in various non-intensive COVID-19 units	38	0	Hospitalised (not further specified): 100%	Laboratory confirmed: 100%	Female 29% Male: 71%	Not reported if pre-existing cognitive impairment present or COVID-19 related neurological symtoms present	NR	12.4 (3.2)	54.5 (12.6)	132.9 (36.6)
Jaywant et al 2021	USA	Single centre	Required rehabilitation but now discharged	No control	April to July 2020	Inclusion (1) hospitalized for acute COVID-19 (2) medically stable but with impairment in mobility and/or activities of daily living necessitating transfer to acute inpatient rehabilitation (3) referred for neuropsychological evaluation for assessment of suspected cognitive dysfunction and to guide rehabilitatio	57	0	Hospitalised (not further specified): 23% ITU (I and V): 77%	Laboratory confirmed: 100%	Female: 25% Male: 75%	Included patients with known cognitive dysfunction (4%)	White: 39% Asian: 19% Black: 12% Hispanic: 28% Mixed race/Other: 2%	NR	64.5 (13.9)	43.2 (19.2)

						n/discharge planning,										
Aiello et al 2021	Italy	Single centre	Discharged or remained in community if never admitted	No control	NR	Data from fifty-four COVID-19-recovered patients referred to ICS Maugeri, IRCCS Pavia (Northern Italy) have been retro-spectively collected	50	0	Mildly symptomatic: 4.5% Mild-to-moderate: 13.6% Moderate-to-severe: 81.8%	NR	Female: 14% Male: 86%	Included patients already at risk of cognitive decline	NR	10.9 (3.1)	66.5 (9.9)	70.5 (34.4)
Henneghan et al 2021	USA	Single Centre	Discharged or remained in community if never admitted	No control	January to February 2021	Adults aged 21–75 years who tested positive for COVID-19, or presumed positive by the medical team, were willing and able to complete remote data collection (cognitive testing; questionnaires) and who spoke English or Spanish were included. Persons with a pre-COVID-1	52	0	Mild illness:56% Moderate illness: 37% Severe illness: 6% Critical illness: 1%	Laboratory confirmed: 100%	Female: 79% Male: 21%	Persons with a pre-COVID-19 diagnosis of significant neurological disorders were excluded.	White: 71% Other: 29%	GED/high school: 6% Associates: 8% Bachelors degree:54% Graduate degree: 15% Some college: 10% Unknown: 7%	37.2 (12.1)	120 (95)

						9 diagnosis of significant neurological disorders were excluded.									
Bonizzato et al 2021	Italy	Single centre	Rehabiliation	No control	NR	met COVID-19 diagnostic criteria and after the acute phase of the disease have been moved to the rehabilitative unit.<br><br>Exclusion criteria were disorders, which pre-cluded answering to the tests, such as delirium, aphasia, or overt dementia.	12	0	Hospitalised (not further specified): 100%	Laboratory confirmed: 100%	Female: 42%<br>Male: 58%	No patients had previous dementia but one had some degree of memory impairmen. Four patients had CVA during acute phase of illness	NR	7.3 (3.3)	71.3 (10.0)	90
Hosp et al 2021	Germany	Single centre	Discharged or rehabilitation centre	No control	April to May 2020	A positive SARS-CoV2 RT-PCR result from nasopharyngeal swabs, age >18 years and presentatio	29	0	Hospitalised (not ITU): 76%<br>ITU (not I and V) 14%<br>ITU (I and V): 10%	Laboratory confirmed: 100%	Female: 32%<br>Male: 68%	Patients presented with new neurological symptoms	NR	13.2 (3.0)	65.2 (14.4)	29.6

						n of at least one newly acquired neurological symptom.										
Manto vani et al 2021	Italy	Single centre	Discharged or remained in community if never admitted	No control	Feb rua ry to Ma y 202 0	(a) age 18–65 years; (b) no history of neurological , cerebrovasc ular, psychiatric dis- orders, or substance use disorders that might interfere with cognition; (c) > 6-month follow-up after SARS-CoV-2 infection; (d) negative nasopharyn geal swab test; and (e) no history of fatigue before SARS-CoV-2 infection.	37	0	Community: 9% Hospitalised (not ITU): 68% ITU (I and V not specified): 23%	Laborat ory confir med: 100%	Female: 34% Male: 66%	no history of neurological , cerebrovasc ular, psychiatric dis- orders, or substance use disorders that might interfere with cognition;	NR	12.9 (3.4)	51.9 (10.9)	6.1 (0.3) months

Rota et al 2021	Italy	Single centre	Rehabilitation	No control	NR	Excluded if visual or hearing impairment, non-native speaker, illiterate, premorbid neurological disease, premorbid critical illness of develop neurological complications during the acute phase of COVID-19	23	0	Hospitalised (not further specified): 39%<br>ITU (I and V): 61%	Laboratory confirmed: 100%	Female: 70%<br>Male: 30%	No patients with pre-exisiting neurological disease included or those that developed neurological sequlae of COVID-19	NR	9 (5)	64 (12)	>42
Hellgren et al 2021	Sweden	Multi centre	Discharged	No control	NR	included all patients (n=734) with a laboratory-confirmed COVID-19 diagnosis admitted to hospital for COVID-19 in the total population of Region Östergötland, Sweden, during the period 1 March to 31 May 2020. Excluded cases with the following	35	0	Hospitalised (not further specified): 43%<br>ITU (not I and V)): 3%<br>ITU (I and V): 54%	Laboratory confirmed: 100%	Female: 20%<br>Male: 80%	No patients with pre-existing cognitive syndromes<br><br>Patients invited for assessment if reporting post COVID-19 symptoms impacting on quality of life	NR	<9: 29%<br>9-12: 42%<br>>12: 29%	59 (51-66) median (IQR)	median (IQR) 142 (120-167)

						characteristics: (1) severe pre-existing comorbidities (such as dementia or under palliative care)										
Gouraud et al 2021	France	Single centre	Discharged	No control	March and April 2020	SARS-CoV-2 infection discharge from tertiary university hospital	100	0	Hospitalised (not further specified): 69%<br>ITU (I and V not stated): 31%	Laboratory confirmed: 100%	Female: 29%<br>Male: 71%	NR	NR	NR	60 (49.5-71.5) median (IQR)	>30
Becker et al 2021	USA	Single centre	Discharged or remained in community if never admitted	No control	April 2020 to May 2021	were 18 years or older, spoke English or Spanish, tested positive for SARS-CoV-2 or had serum antibody positivity, and had no history of dementia.	740	0	Community: 51%<br>ED only: 22%<br>Hospitalised (not further specified): 27%	Laboratory confirmed: 100%	Female: 63%<br>Male: 37%	No known history of dementia	White: 54%<br>Asian: 28%<br>Black: 15%<br>Hispanic: 20%<br>Mixed race/Other: 11%	≤12: 14%<br>>12: 86%	49.0 (14.2)	7.6 (2.7) months
Albu et al 2021a	Spain	Single-centre	Post-ITU and Non-ITU	No control	NR	Inclusion: adults >18 years with persistent symptoms COVID-19 Exclusion criteria were: previous neurological	30	0	ITU (not further specified): 54%<br>Hospitalised (not further specified): 46%	Laboratory confirmed: 100%	Male: 63%<br>Female:37%	Presenting with persistent symptom. Excluded pre-existing comorbidities	NR	NR	54 (43.8–262)	103 (93–116)

						, psychiatric or severe medical condition; persistent symptoms of confirmed COVID-19 without indications for rehabilitation										
Di Pietro et al 2021	Italy	Single-Centre	Rehabilitation	No control	May–September 2020.	Inclusion criteria: patients who needed, besides the rehabilitation program, an extensive neuropsychological evaluation during hospitalization; diagnosis of COVID-19 infection Exclusion criteria: patients with delirium present or antipsychotic therapy	12	0	ITU (I and V): 100%	Laboratory confirmed: 100%	Male: 58% Female:42%	Not excluded if pre-existing cognitive impairment. Presented with severe rehabilitation needs.	NR	8.125	64.0 ± 13.7	>30

Negrini et al 2021	Italy	Single centre	Discharged	No control	March 3 and April 8, 2020	NR	9	0	Hospitalised (not ITU): 45%<br>ITU (I and V): 55%	Laboratory confirmed: 100%	Male: 66.7%<br>Female: 33.3%	No exclusion criteria	NR	10 (5-18y range)	60 (21-77y range)	42.3
Albu et al 2021b	Spain	Single centre	Patients referred to 8 weeks of multidisciplinary rehabilitation	NR	June and December 2020	Inclusion criteria were: adults >18 years with neurological, cog-nitive and musculoskeletal sequelae and persistent symptoms of COVID-19 infection (>3 months after initial symptoms) confirmed by either PCR or serology. Exclusion criteria were: previous neurological, psychiatric or severe medical condition	43	0	Community: 23%<br>Hospitalised (not ITU): 21%<br>ITU (I and V not stated): 56%	Laboratory confirmed: 100%	Male: 55.8%<br>Female: 44.2%	Presenting with persistent symptoms but with no pre-existing cognitive impairment or other co-morbidities	NR	NR	52 (11.4)	130 ± 48 days

Bouts et al 2021	Belgium	Multicentre	Discharged	NR	March to June 2021	COVID-19 ARDS survivors, admitted to ITU during 2020 spring outbreak	45	0	ITU (I and V): 100%	Laboratory confirmed: 100%	Male: 64% Female: 36%	No exclusion criteria	NR	NR	63(11)	6 months
Henrie et al 2021	France	Single centre	Discharged	NR	NR	All ARDS patients admitted to ITU	10	0	ITU (not further specified): 100%	NR	Male: 80% Female: 20%	No exclusion criteria	NR	NR	Mean, range 60 (52-73)	3 months
Dondaine et al 2022	France	Single centre	Discharged	No control	September 2020 to December 2021	inclusion: a diagnosis of COVID-19 confirmed with subjective cognitive complaints Exclusion: (i) severe pneumonia following COVID-19 (ii) the presence of asthma, unstable coronary heart disease, uncontrolled diabetes or hypertension, encephalitis, or epilepsy; (iii) a history of head injury or a brain	62	0	ITU (not further specified): 50% Community: 50%	Laboratory confirmed: 100%	ITU Male: 52% Female: 48% Community Male: 32% Female: 68%	Included patients with post COVID-19 subjective cognitive complaints. Excluded patients with significant comorbidities	NR	ITU 14.35 (2.56) Community 14.84 (2.24)	ITU 52.19 (8.72) Community 43.90 (10.92)	3-9 months

						tumor; (iv) the presence of a major psychiatric condition (v) dementia									
Voruz et al 2022	Switzerland	Single centre	Discharged	No control	March 2020 to May 2021	Inclusion: Patients who had an infection confirmed by PCR Exclusion: A history of neurological issues, psychiatric disorders, cancer and neurodevelopmental pathologies,	121	0	ITU (I and V): 20% Hospitalised (non-ITU): 40% Community: 40%	Laboratory confirmed: 100%	Male: 68% Female: 32%	Excluded patients with history of neurological disorders and other significant comorbidities	NR	Mean education level: 2.65 (0.54)	56.69 (10.41)	222.46 (42.93)
Ferrucci et al 2022	Italy	Single centre	Discharged	No control	NR	Patients hospitalised with COVI-19 >18 years old	76	0	ITU (I and V): 9 % Hospitalised (not otherwise specified): 91%	Laboratory confirmed: 100%	Male: 74% Female: 26%	55% of patients reported subjective cognitive impairment	NR	<8 years: 29% 9-13 years 43%: >13 years: 28%	56.24 (12.1)	5 months and 12 months

De Pemille et al 2022	France	Single centre	Discharged	No control	April 2021 to May 2021	Inclusion: > Admission to the ITU for SARS due to COVID-19 with need for mechanical ventilation with tracheal intubation between discharged to a post-ITU medical unit within our institution.<br>Exclusion: prolonged stuporous state after ITU discharge; focal neurological deficit to avoid any potential associated acute vascular disease	13	0	ITU (I and V): 100%	Laboratory confirmed: 100%	Male: 62%<br>Female: 38%	Excluded patients with neurological complications. No patient had a pre-ITU cognitive complaint	NR	Secondary school or higher: 69%<br>Primary school: 31%	Median (IQR): 62 (51-68)	3 months

Delgado-Alonso et al 2022	Spain	Single centre	Discharged	No control	NR	Inclusion: Cognitive complaints temporally related with the SARS-CoV-2 infection.. Exclusion: 1) Any cognitive complaint before COVID-19; 2) History of any neurological disorder potentially associated with cognitive impairment; 3) Active psychiatric disorder or previous psychiatric disease	50	0	ITU (I and V): 8%<br>ITU (not I and V): 2%<br>Hospitalised (non-ITU): 26%<br>Community: 64%	Laboratory confirmed: 100%	Male: 26%<br><br>Female: 74%	Attending clinic for new cognitive issues. Excluded patients with pre-existing neurological or psychiatric conditions	NR	13.58 (4.01)	51.06 (11.65)	9.12 (3.46) months
Braga et al 2022	Brazil	Single centre	Discharged	No control	April 2021 to January 2022	Inclusion: COVID-19 survivors who sought treatment for cognitive issues<br>Exclusion:<br>a) cognitive decline, stroke, TBI, and any other neurological	614	0	ITU (I and V): 10.6%<br>ITU (not I and V): 6.7%<br>Hospitalised (not ITU): 16%<br>Community: 66.6%	Laboratory confirmed: 100%	Male: 27%<br><br>Female: 73%	Patients seeking treatment for cognitive issues. However, excluded pre-existing neurological	NR	5-8: 5%<br>9-11: 6%<br>12-15: 35%<br>16+: 54%	18-39: 23%<br>40-59: 62%<br>60+ 15%	8 months (4.3)

						condition with compromised cognitive function existent before the COVID-19 diagnosis, b) severe past depression									
Garcia-Sanchez et al 2022	Spain	Single centre	Discharged	No control	July 2020 to May 2021	Inclusion: (a) having had COVID-19-19 and referred for neuropsychological assessment after reporting subjective cognitive complaints; and. Exclusion: documented medical history of neurological or psychiatric conditions before the infection	63	0	ITU (not further specified): 23.8% Hospitalised (not ITU): 28.5% Community: 47.7%	Laboratory confirmed: 100%	Male: 37% Female: 63%	Presenting with cognitive complaints. Excluded patients with existing neurological or psychiatric conditions	NR	14.4 (3.1)	51.1	187 (99)

Schindler et al 2022	USA	Single centre	Discharged	No control	NR	Patients presenting to the University of Pennsylvania Neuro-COVID-19 Clinic (PNCC)	94	0	ITU (not further specified): 4%<br>Hospitalised (not ITU): 26%<br>Community: 70%	NR	Male: 33%<br><br>Female: 67%	Presenting with subjective cognitive complaints to a specialist clinic	NR	N9%R	Mean (range): 50 (21-75)	Mean (range) 234 (40-509)
Lauria et al 2022	Italy	Single centre	Discharged	No control	April 2020 to November 2020	Patients presenting to post-acute outpatient service for patient's recovering from COVID-19 and over 65 years of age	100	0	ITU (I and V): 15%<br>Hospitalised (not ITU): 73%<br>Community: 12%	Laboratory confirmed: 100%	Male: 65%<br><br>Female: 35%	Presenting for post COVID-19-19 clinic. No exclusion criteria	NR	12.7 (8.7)	73.4 (6.1)	96.5 (45.3)
Jennings et al 2022	Ireland	Single centre	Discharged	No controls	NR	Inclusion: aged 18 years or older; (ii) a self-reported history of SARS-CoV-2 infection; (iii) experiencing prolonged symptoms such as fatigue; (iv) able to mobilise independently, with or without an aid; (v) able to transfer	108	0	ITU (not further specified): 3.7%<br>Hospitalised (not ITU): 17.6%<br>Community: 78.7%	Laboratory confirmed: 100%	Male: 29%<br><br>Female: 71%	Experiencing post COVID-19 fatigue. No exclusion of pre-existing conditions	NR	Completed third-level education: 69%<br>NR: 31%	46.3 (10.3)	324.4 (184.5)

						independently or with minimal assistance of one person from a lying to standing position; and (vi) able to provide informed consent.									
Ferrando et al 2022	USA	Multi centre	Discharged	No control	NR	Exclusion: Persons with a prior diagnosis of a major neurocognitive disorder, traumatic brain injury with loss of consciousness, uncorrected visual/hearing deficits, intellectual disability, or unstable psychiatric symptoms	60	0	Hospitalised (not ITU): 12% Community: 88%	Laboratory confirmed: 100%	Male: 32% Female: 68%	Excluded patients with known neurocognitive disorder. Included patients seeking help for subjective cognitive complaints	NR	16.0	41.4 (13.5)	209.3 (133.5)
Bungenberg et al 2022	Germany	Single centre	Discharged	No control	August 2020 to March 2021	Patients with COVID-19 presenting with persisting symptoms to outpatient clinics	50	0	Hospitalised (not ITU): 20% ITU (not further specified): 22% Community: 58%	Laboratory confirmed: 100%	Male: 44% Female 56%	Presenting with persistent symptoms. No exclusion criteria	NR	15.5	50.5	29.3 weeks

Voruz et al 2022	Switzerland	Multicentre	Discharged	No control	NR	Inclusion: Recruited via admission lists or from COVID-19-COG cohort with confirmed COVID-19 infection 6-9 months prior<br>Exclusion: history of neurological or psychiatric disorders cancer, neurodevelopmental pathologies, pregnancy and age above 80 years.	102	0	Hospitalised (not ITU): 33%<br>ITU (I and V): 23%<br>Community: 54%	Laboratory confirmed: 100%	Male: 44%<br>Female 56%	Excluded if history of neurological or psychiatric disorder	NR	Level of education 2.68 (0.50)	56.49 (9.6)	230.25 (43.83)
Calabria et al 2022	Spain	Single centre	Discharged	No control	July 2020 to January 2020	Inclusion: referred for neuropsychological assessment after reporting subjective cognitive complaints; and being 18 + years old.<br>Exclusion: a documented medical	136	0	Hospitalised (not ITU): 26%<br>ITU (not further specified): 18%<br>Community: 56%	Laboratory confirmed: 100%	Male: 36%<br>Female 64%	Presenting with subjective cognitive complaints. Excluded if history of neurological or psychiatric disorder	NR	13.6 (3.2)	51.7 (13.5)	252 (149)

						history of neurological or psychiatric conditions before the infection.										
Krishnan et al 2022	USA	Single centre	Discharged	No control	September 2020 and April 2021	Inclusion: Patients over 18 years old referred for neuropsychological assessment due to subjective post COVID-19 cognitive complaints Exclusion: major pre-existing neurological conditions and suboptimal task engagement	20	0	Hospitalised (not ITU): 25%<br>ITU (not I and V): 5%<br>ITU (I and V): 10%<br>Community: 60%	Laboratory confirmed: 100%	Male: 10%<br>Female 90%	Presenting with subjective cognitive complaints. Excluded if history of neurological or psychiatric disorder	White: 70%<br>NR: 30%	15.2 (2.6)	45	168 (69.3)
Whiteside et al 2022	USA	Single centre	Discharged	No control	November 2020 and June 2021	Inclusion: outpatients diagnosed with COVID-19 who were referred for a neuropsychological evaluation for clinical/treatment	49	0	Hospitalised (not ITU): 4%<br>ITU (not I and V): 8%<br>ITU (I and V): 18%<br>Community: 70%	Laboratory confirmed: 100%	Male: 16%<br>Female: 84%	Presenting with subjective cognitive complaints. No exclusion criteria	White: 80%<br>Black: 4%<br>Latino: 4%<br>Asian: 2%	14.47 (2.16)	49.65 (12.43)	197.47 (53.20)

						planning purposes in the context of cognitive concerns following COVID-19 infection										
Damiano et al 2022	Brasil	Single centre	Discharged	No control	March 2020 and September 2020	Inclusion: All patients discharged from hospital after treatment for COVID-19 at single centre Exclusion: Pre-existing dementia or severe intellectual disability	424	0	Hospitalised (not ITU): 51.5% ITU (not I and V): 19.4% ITU (I and V): 30.1%	Laboratory confirmed: 98.6% High clinical suspicion/CT: 1.4%	Male: 52% Female: 48%	Excluded if pre-existing dementia or severe intellectual disability	NR	Nil: 4.5% Incomplete elementary school: 33.4% Elementary school: 11.1% Incomplete high school: 6.6% High school: 27.8% Incomplete bachelor: 4.7% Bachelor: 8.0% Post-graduation: 4%l	55.7 (14.2)	207 (20.4)
Holdsworth et al 2022	UK	Single centre	Discharged	No control	August 2020 and April 2021	NR	205	0	ED and discharged: 45% Hospitalised (not ITU): 21% ITU (not I and V): 2% ITU (I and V): 2.9% Community: 29.1%	Laboratory confirmed: 68% High clinical suspicion/CT: 32%	Male: 83.4% Female: 16.6%	No exclusions	White: 83.3% BAME: 16.7%	NR	38.3	Median (range) 24 weeks (17.1-34.0)

Costas-Carrera et al 2022	Spain	Single centre	Discharged	No control	April 2020 to December 2020	Adult patients who were admitted to the Intensive Care Unit (ITU) for SARS-CoV-2 infection.<br><br>Exclusion: insufficient language proficiency, terminal disease and previous neurodegenerative disease	58	0	ITU (I and V not specified): 100%	Laboratory confirmed: 100%	Male: 71% Female: 29%	Excluded patients with pre-existing neurodegenerative conditions	NR	Primary: 8.6% Secondary: 21% Graduate/Postgraduate level: 69% Unknown: 1.4%	67 (9)	180
Mazza et al 2021	Italy	Single centre	Discharged	No control	April 2020 to June 2020	Inclusion: Clinical and radiological findings suggestive of COVID-19 pneumonia at the admission to the Emergency Department with confirmed PCR.<br><br>Exclusion: Under 18	226	0	Hospitalised (not further specified): 78% ED and discharged: 22%	Laboratory confirmed: 100%	Male: 66% Female: 34%	Did not exclude patients with pre-existing neurological, cognitive or psychiatric conditions	NR	12.5 (4)	59 (13)	90

Huang et al 2022	China	Single	Discharged	Healthy controls	February 2020 – April 2020	COVID-19 diagnosis, discharged between feb and april 2020, >18 years of age, willingness and ability to undergo MRI scanning<br><br>Exclusion: Structural abnormality on MRI, severe psychiatric disease, severe somatic disease, drug abuse, history of TBI or surgery or brain structural abnormalitye	22	21	Hospitalised (not ITU): 64%<br>ITU (I and V not stated): 36%	Laboratory confirmed: 100%	Male: 50%<br>Female: 50%	Excluded if previous significant comorbidity. <50% presenting with non specific neurological sequalae	NR	Post COVID-19: 12 (12-16)<br><br>Controls: 12 (10.5-16)	54.14 (9.76)	Median (IQR) 351.5 (329.8-357.3)

Lamontagne et al 2021	Canada	NR	Remained in community if never admitted	Healthy controls	January 2021 to March 2021	Inclusion: age between 18 and 60 years, fluency in English and, for the COVID-19 group, a past COVID-19 diagnosis Exclusion: Reports of mood irregularities, cognitive deficits; lifetime history of chronic or serious medical, neurological or hormonal disturbances, current or past alcohol/drug abuse, or any psychological disorder	47	50	Community: 100%	Laboratory confirmed: 100%	Post COVID-19 Male: 42% Female: 58%<br><br>Control Male: 30% Female: 70%	Excluded if significant comorbidty of cognitive impairment.	Post COVID-19: White: 52% Asian: 28% Black: 20% Hispanic: 16% Mixed race: Other: 4%<br><br>Control: White: 52% Asian: 10% Black: 16% Hispanic: 16% Mixed race: Other: 6%	Post COVID-19: 16.1 (3)<br><br>Control: 15.5 (3)	Post COVID-19 30.80 (7.79)<br><br>Control: 29.14 (9.87)	123.63 (94.71)

Zhao et al 2022	UK	Single centre	Remained in community if never admitted	Healthy controls	NR	Exclusion: Admitted to hospital for COVID-19 COVID-19 symptoms impacted daily life Had/having severe long-COVID-19 symptoms	53	83	Community: 100%	Laboratory confirmed: 75% Clinically suspected: 25%	Post COVID-19 Male: 60% Female: 40%<br><br>Control Male: 63% Female: 37%	Proportion of patients presenting with residual symptoms. No exclusion criteria	NR	No significant difference between cohorts	Post COVID-19: 28.0 (8.6)<br><br>Control 29.0 (10.3)	163.0 (128.1)
Versace et al 2021	Italy	Single centre	Rehabilitation	Healthy controls	NR	inclusion criteria were: a) absence of neurological dis- orders prior to COVID-19, b) absence of prior or current diagnosis of conditions related to fatigue, c) absence of dyspnoea or other long-lasting sequelae COVID-19 d) absence of anaemia, e) no treatment with corticosteroids,	12	10	ITU (I and V not specified): 100%	Laboratory confirmed: 100%	Post COVID-19 Female 83% Male: 17%<br><br>Control Male: 80% Female: 20%	Treated for neurological complications of COVID-19 but with no pre-existing impairments	NR	Post COVID-19: 11.8 (3.5)<br><br>Control: 12.8 (3.8)	Post COVID-19: 67 (9.6)<br><br>Control: 61 (8.2)	63-91 range

						antihistaminic, antihypertensive, diuretic, or hypnotic drugs at the time of study.									
Miskowiak et al 2021	Denmark	Single centre	Discharged	Healthy controls	March to June 2020	Patients presenting to a post COVID-19 respiratory clinic	29	100	Hospitalised (not further specified): 100%	Laboratory confirmed: 100%	Post COVID-19: Female: 41% Male: 59%<br><br>Control: Female:59% Male: 41%	patients were excluded due to substantial language barriers or disabilities.	NR	Post COVID-19: 14.3 (3.9)<br><br>Control: 14.3 (3.0)	Post COVID-19: 56.2 (10.6)<br><br>Control: 56.0 (6.9)	90-120
Hampshire et al 2021	UK	Multicentre	Discharged or remained in community if never admitted	Healthy controls	January to December 2020	Participants able to undertake a clinically validated web-optimized assessment, and questionnaire items capturing self-report of suspected and confirmed COVID-19 infection and respiratory symptoms.	12689	68648	Community: 98.5% Hospitalised (not ITU): 1.2% ITU (I and V): 0.3%	Laboratory confirmed: 3% Clinically suspected: 97%	Female: 55% Male: 45%	Included patients with previous psychiatric conditions and residual symptoms<br><br>Analysis of markers of premorbid intelligence did not support differences being present prior to infection.	White: 92.2% Asian: 3.3% Black: 0.3% Hispanic: 0.4% Mixed race/Other: 3.8%	No schooling: 0.1% Primary/elementary school: 1.9% Secondary school/high school: 35.4% University degree:58.6% PhD: 4.0%	46.7 (15.7)	Range 30 - 270 days

Ortelli et al 2021	Italy	Single centre	Rehabilitation	Healthy controls	April and May 2020	Inclusion criteria were a) almost total resolution of the neurological symptoms resulting from COVID-19, b) FRS score ≥ 6, c) absence of neurological disorders prior d) absence of prior or current conditions related to fatigue, e) absence of dyspnoea or other long-lasting sequelae of interstitial COVID-19 pneumonia, no treatment with corticosteroids, antihistaminic, antihypertensive, diuretic, or hypnotic drugs at the	12	12	ITU (not further specified: 100%	Laboratory confirmed: 100%	Male : 83% Female:17%	No pre-existing symptoms but patients requiring rehabilitation post COVID-19.	NR	11.83	67 ± 9.6	11.5 weeks

						time of study.									
Mattioli et al 2021	Italy	Single centre	A group of HCW who had been previously affected by COVID-19	Healthy controls	By end of February 2020	NR	120	30	Community: 100%	Laboratory confirmed: 100%	Post COVID-19: Male: 25% Female, 75%<br><br>Controls: Male: 24% Female, 76%	No exclusion criteria	NR	Post COVID-19: 16 (8–18) Controls: 18 (8–18)	Post COVID-19: 47.86 (26–65) Controls: 45.73 (23–62)	125.92 (12–215) weeks
Poletti et al 2022	Italy	Single centre	Discharged	Healthy controls	May 2020 and February 2021	Inclusion criteria for COVID-19 survivors were clinical and radiological findings suggestive of COVID-19 pneumonia at the admission to the Emergency Department . excluded from the study if they presented intellectual disabilities or neurological disorders	312	165	Hospitalised (not further specified): 100%	Laboratory confirmed: 100%	Post COVID-19: Male 75% Female 25%<br><br>Control: Female: 42% Male: 58%	Excluded if presented with intellectual disabilities or neurological disorders	NR	Post COVID-19 12.94±3.76 Control:13.45±3.79	Post COVID-19 52.63 (8.81) Control :40.57 (11.79)	1,3,6-months

Miskowiak et al 2022	Denmark	Single centre	Discharged	Healthy controls	March to July 2020	all adult patients (≥ 18 years) admitted with COVID-19 to Bispebjerg Hospital in Denmark	25	55	Hospitalised (not further specified): 100%	Laboratory confirmed: 100%	Post COVID-19: Male: 52% Female:48%<br><br>Control Male: 54% Female: 46%	No exclusion criteria	Caucasian: 75%<br><br>Other: 25%	Post COVID-19 14.84 (3.8)<br><br>Control: 14 (2.7)	Post COVID-19 56 (10.7)<br><br>Control: 56.7 (5.2)	12 months
Serrano-Castro et al 2022	Spain	Multicentre	Discharged	Healthy controls	NR	Inclusion: Respiratory failure with criteria for hospital admission; radiological criteria for lung disease More than 90 days and less than 120 days since hospital discharge Exclusion: Cognitive impairment Motor, sensorial, or intellectual disability or illiteracy that prevented performing tests.	46	40	Hospitalised (not further specified): 100%	Laboratory confirmed: 100%	Post COVID-19: Male: 37% Female: 63%<br><br>Control Male: 50% Female: 50%	Excluded patients with cognitive impairment or those e with Motor, sensorial, or intellectual disability or illiteracy	NR	NR	Post COVID-19 71 (10.1)<br><br>Control: 52.2 (2.3)	90-120 (range)

Ollila et al 2022	Finland	Single centre	Discharged	Healthy controls	March 2020 and December 2020	Inclusion: Adults aged 18 years or older with a confirmed (reverse transcription-polymerase chain reaction or antibody testing) SARS-CoV-2 Only subjects fluent in Finnish were eligible. Only patients with complete neuropsychological assessment data were included in the present study<br><br>Exclusion: prior major neurological diagnosis (traumatic brain injury, dementia, stroke, Parkinson's disease), developmental disability, or	165	48	ITU (not further specified): 51%<br><br>Hospitalised (non-ITU): 35%<br><br>Community: 31%	Laboratory confirmed: 100%	Post COVID-19: Male 49% Female: 51%<br><br>Control Male: 52% Female: 48%	Excluded patients with prior major neurological diagnosis (traumatic brain injury, dementia, stroke, Parkinson's disease), developmental disability, or substantially impaired hearing or vision	NR	ITU: 13.6 (2.7) Hospitalised: 14.9 (2.7) Community: 15.6 (2.1) Control: 15.4 (2.9)	Median IQR ITU: 59 (49-65.3) Hospitalised: 57 (49-62) Community: 44.5 (34.3-52) Control: 56 (49-63.3)	209 (25)

						substantially impaired hearing or vision.									
Andriuta et al 2022	France	Single centre	Discharged	Healthy controls	NR	Inclusion: French-speaking patients with a post-acute COVID-19 cognitive complaint referred to a memory centre Exclusion: 1) illiteracy, 2) alcoholism or severe comorbidities 3) concurrent neurological and/or psychiatric disorders and 4) a history of major or minor NCD	46	1003	ITU (not further specified): 24%<br><br>Hospitalised (non-ITU): 37%<br><br>Community: 39%	Laboratory confirmed: 100%	Post COVID-19: Male: 23.9% Female 76.1%<br><br>Controls Males: 35.9% Female: 64.1%	Excluded if significant comorbidity but presenting with cognitive complaint	NR	Post COVID-19: Primary: 8.7% Secondary: 30.4% Tertiary: 60.9%<br><br>Controls: 11.4 (3.2)	Post COVID-19: 50.9 (14)<br><br>Controls: 62 (11.3)	254 (90)

Morelli et al 2022	USA	Single centre	Mixture of Discharged to rehabilitation and long-term care	Patients with COPD/ILD	August 2020 to July 2021	Inclusion: Patients with critical or severe COVID-19 attending outpatient follow up clinc<br>Exclusion: an acute or chronic neurologic, neurodegenerative, or orthopedic condition or disease that influenced cognition or motor performance	56	36	ITU (I and V): 64%<br><br>Hospitalised (non-ITU): 36%	Laboratory confirmed: 100%	ITU<br>Male: 59%<br>Female: 41%<br><br>Hospitalised: Male: 50%<br>Female: 50%<br><br>Control<br>Male: 49%<br>Female: 51%	Excluded if acute or chronic neurological or neurodegenerative condition	ITU<br>White: 49%<br>Black: 38%<br>Hispanic: 14%<br><br>Hospitalised: 70%<br>Black: 20%<br>Hispanic: 10%<br><br>Control 76%<br>Black: 22%<br>Hispanic: 2%	NR	ITU<br>55.7 (12)<br><br>Hospitalised: 57.7 (11)<br><br>Control 66.2 (10.4)	30 and 90
Cecchetti et al 2022	Italy	Single centre	Discharged	Healthy control	April and May 2020	Inclusion:<br>Patients evaluated at the 1-month post-discharge neurological examination<br>Exclusion: medical illnesses or substance abuse that could interfere with cognitive functioning; any (other) major	49	36	ITU (not further specified): 4.1%<br>Hospitalised (not ITU): 95.9%	Laboratory confirmed: 100%	Male: 75%<br><br>Female: 25%	Patients presented with self reported cognitive complaints. However, patients with medical illnesses or substance abuse that could interfere with cognitive functioning	NR	11.3 (3.9)	60.6 (12.9)	2 and 10 months

						systemic, psychiatric, or neurological illnesses; and other causes of focal or diffuse brain damage at routine MRI										
Hampshire et al 2022	UK	Single centre	Discharged	Healthy controls and those with dementia	March 2020 and July 2020	All patients admitted to Hospital with COVID-19 between who survived and consented to take part were eligible for this cohort study	46	120	Hospitalised (not further specified): 65%<br>ITU (I and V): 35%	NR	Male: 42%<br><br>Female: 58%	Nil exclusion criteria	NR	<College: 9%<br>College:28%<br>University:37%<br>NR/Other: 26%	51 (14)	179 (62)

Ortelli et al 2022a	Italy	Single centre	Discharged	Healthy controls	January 2021 and March 2021	Inclusion: Previous PCR positive, mild form of COVID-19 disease, complaints of cognitive diffITUlties/ sense of fatigue.<br>Exclusion: prior or concurrent diagnosis of neurological, psychiatric, endocrine, metabolic or cardiopulmonary conditions; (b) clinical and/or radiological evidence of COVID-19 related pneumonia during the active phase of the disease; (c) anaemia; (d) current pharmacological treatment with corticosteroids,	67	22	Community: 100%	Laboratory confirmed: 100%	Male: 25.5%<br><br>Female: 74.5%	Presenting to specialist clinic with self-reported fatigue/cognitive impairment. Excluded patients with conditions or prescriptions that may impact cognitive outcomes	NR	COVID-19: 14.1 (2.7)<br><br>Control: 14.3 (2.7)	COVID-19: 49.7 (13.3)<br><br>Control: 46.4 (14.2)	109 (77)

						antihistamines, antihypertensives, diuretics, antidepressants, anxiolytic or hypnotic drugs at the time of study									
Crivelli et al 2022	Brasil	Multi-centre	Discharged	Healthy controls	NR	Inclusion criteria were: a positive SARS-CoV2 RT-PCR result from nasopharyngeal swabs, age > 18 years, and no pre-infection cognitive complaint. Exclusion criteria were: significant upper limb impairment, visual acuity or visual field deficits, drug use, or psychiatric disorders	45	45	Hospitalised (not further specified): 31% Community: 69%	Laboratory confirmed: 100%	Male: 56%<br>Female: 44%	Attending outpatient neurology clinics. No pre-existing cognitive complaints	NR	COVID-19: 17<br>Control: 17	COVID-19: 50<br>Control: 57	142

Appelt et al 2022	Brasil	Multi-centre	Discharged	Healthy controls	September 2020 and September 2021.	Included: mild to moderate COVID-19 symptoms who met the COVID-19 diagnostic standard, had an education level greater than nine years, and could complete the tests independently<br>Excluded: Patients with severe and critical COVID-19, history of illness or medication that may interfere with assessment	53	30	Hospitalised (not further specified)/ Community: 100%	Laboratory confirmed: 100%	Male: 35%<br><br>Female: 65%	No pre-existing cognitive complaints	COVID-19<br>White: 79.2%<br>Black: 16.9%<br>Asian: 3.7%<br><br>Control<br>White: 80%<br>Black: 16.7%<br>Asian: 6.7%	Median (IQR)<br>COVID-19: 14.3 (11-21)<br><br>Control: 14.8 (10-22)	Median (IQR)<br>COVID-19: 42.3 (25-69)<br><br>Control: 37.9 (21-55)	3-6 months and 6-12 months
Shanley et al 2022	USA	Multicentre	Discharged	Patients with pre-existing neurological conditions	October 2020 to October 2021	Inclusion: either preexisting or new neurological condition, positive COVID-19 test or high clinical likelihood	40	16	Hospitalised (not ITU): 7.5%<br>Community: 92.5%	Laboratory confirmed: 97.5%<br>Clinically suspected: 2.5%	Male: 32%<br><br>Female: 68%	Mixture of patients with pre-existing and new neurological complaints	NR	NR	50.5 (26.25)	Weeks 16.1

Rubega et al 2022	Italy	Single centre	Discharged	Healthy controls	March 2020 and May 2020	Inclusion: Discharged from ITU and medical wards after treatment for COVID-19- Exclusion: age < 18 years, previous diagnosis of cognitive impairment, previous diagnosis of neurological disorder, on drugs altering sleep architecture	33	12	Hospitalised (not ITU): 52%<br>ITU (not further specified): 48%	NR	Male: 73%<br><br>Female: 27%	Excluded if pre-existing neurological or cognitive condition	NR	NR	Range 49-80	12 months
Ortelli et al 2022b	Italy	Single centre	Discharged	Healthy controls	March 2021 and July 2021	Patients with post COVID-19 neurological symptoms with a mild form of acute disease and persistent cognitive or fatigue type symptoms	74	29	Community: 100%	Laboratory confirmed: 100%	Post COVID-19: Male: 41%<br>Female: 59%<br><br>Control Male: 28%<br>Female: 72%	Patients with post COVID-19 neurological symptoms. Excluded pre exisitng conditions that may impact assessment	NR	COVID-19: 14.3 (2.7))<br><br>Control: 14.8 (2.4)	COVID-19: 48.4 (12.6)<br><br>Control: 44.2 (14.5)	> 3 months

Sneller et al 2022	USA	Single centre	Discharged	Healthy controls	June 2020 to July 2021	Patients recovered from COVID-19 recruited from a single centre	189	122	Hospitalised (not further specified): 11.6%<br>Community: 88.4%	Laboratory confirmed: 100%	COVID-19<br>Male: 45%<br>Female: 55%<br>Control<br>Male: 44.5%<br>Female: 55.5%	104 of 189 patients had persistent symptoms<br>No exclusions.<br>Patients had pre-existing conditions	COVID-19<br>White: 78.3%<br>Other 21.7%<br>Control:<br>White: 70%<br>Other: 30%	NR	COVID-19: 50<br>Control: 51	162

Supplementary Table 3 – GRADE Assessment of Evidence for Prognostic Marker Association with Domain Specific Outcomes

**№ of studies**	**Certainty assessment**						**Effect**		**Certainty**	**Interpretation**
	**Study design**	**Risk of bias**	**Inconsistency**	**Indirectness**	**Imprecision**	**Other considerations**	**№ of individuals**	**Rate (95% CI)**		
**Severity – Executive Function** (Measured by hospitalisation, ICU admission, severity of respiratory symptoms, length of intubation)										
5	observational studies and prospective studies	not serious	moderate	moderate	Not serious	Some unclear reporting of direct comparisons/ANOVA	1164<br><br>305 in high-quality papers	[Spearman's rho=0.44, p=0.02. Spearman's rho=0.64, p<0.001]<br><br>[Chi squared=-1.965, p=0.071]<br><br>[p=0.04]	⊕⊕◯◯ Low	One of two low-quality studies identified correlation between severity and poorer executive function scores.<br><br>One high-quality study identified a correlation between severity of

№ of studies	Certainty assessment						Effect		Certainty	Interpretation
	Study design	Risk of bias	Inconsistency	Indirectness	Imprecision	Other considerations	№ of individuals	Rate (95% CI)		
										illness (respiratory symptoms) and poorer executive function (Trail Making B and Word Fluency Test).<br><br>One high quality study identified a non-significant trend towards poorer performance in executive function (Word fluency test) in those admitted to ICU when compared to those not admitted to ICU.<br><br>One high-quality paper identified an association between ICU admission and better performance on executive function task Trail

№ of studies	Certainty assessment						Effect		Certainty	Interpretation
	Study design	Risk of bias	Inconsistency	Indirectness	Imprecision	Other considerations	№ of individuals	Rate (95% CI)		
										making B, when compared to those not admitted to ICU.
**Severity – Learning and Memory** (Measured by hospitalisation, ICU admission, oxygen requirement, self-reported symptom severity)										
6	observational studies and prospective studies	not serious	moderate	moderate	Not serious	Some unclear reporting of direct comparisons/ANOVA. One study used memory decrement in the learning and memory task as measure.	1097	[AOR (95%CI) 2.2 (1.3-3.8)], [Mean difference p=0.007], [F(1,6)=15.3, p=0.008], [Correlation Coefficient r=0.404, p=0.027], [ChiSquared=0.589, p=0.556], [z=-2.23, p=0.03], [ChiSquared=2.9, p<0.01], [p=0.327]	⊕⊕⊕◯ Moderate	Five of six studies identified correlation between severity and poorer learning and memory scores.

№ of studies	Certainty assessment						Effect		Certainty	Interpretation
	Study design	Risk of bias	Inconsistency	Indirectness	Imprecision	Other considerations	№ of individuals	Rate (95% CI)		
**Severity – Perceptual Motor Function** (Measured by hospitalisation, ICU admission)										
2	observational studies	not serious	Not serious	moderate	not serious	none	822<br><br>45 in high quality study.	[AOR (95%CI) 1.4 (0.8-2.5)]<br><br>[p=0.08]	⊕⊕◯◯ Low	One low-quality study identified a correlation between severity and poorer perceptual motor function scores.<br><br>One high-quality study found a non-significant trend towards association with better scores on Trail Making A in those admitted to ICU, when compared to those not admitted to ICU.

№ of studies	Certainty assessment						Effect		Certainty	Interpretation
	Study design	Risk of bias	Inconsistency	Indirectness	Imprecision	Other considerations	№ of individuals	Rate (95% CI)		
**Severity – Language** (Measured by hospitalisation, ICU admission)										
3	observational studies	not serious	Not serious	moderate	not serious	none	844	[AOR (95%CI) 3.0 (1.7-5.2)], [ChiSquared=-1.965, p=0.071], [ChiSquared=3.5, p<0.01]	⊕⊕⊕◯ Moderate	Three of three studies identified correlation between severity and poorer language scores.
**Severity – Complex Attention** (Measured by hospitalisation, oxygen requirement, respiratory distress)										
3	observational studies and prospective studies	not serious	Not serious	moderate	not serious	none	211<br><br>85 in high-quality paper	[ANOVA F=3.748, p=0.021], [z=3.52, p=0.001]<br><br>[ρ=− 0.43, p=0.03]	⊕⊕⊕◯ Moderate	Three of three studies identified correlation between severity and poorer complex attention scores.<br><br>One of these papers was

№ of studies	Certainty assessment						Effect		Certainty	Interpretation
	Study design	Risk of bias	Inconsistency	Indirectness	Imprecision	Other considerations	№ of individuals	Rate (95% CI)		
										considered high-quality, and found an association between severity as measured by respiratory distress on NEWS score, and complex attention as measured by the Symbol Digit Modalities Test.
**Age – Executive Function** (Measured as age quartiles, and age as continuous variable)										
2	observational studies and prospective studies	not serious	moderate	moderate	Not serious	none	217	[ANOVA F=4.63, p =0.006],[Effect Size 0.247, p<0.001]	⨁⨁◯◯ Low	Two of two studies identified correlation between increasing age and poorer executive function scores.

№ of studies	Certainty assessment						Effect		Certainty	Interpretation
	Study design	Risk of bias	Inconsistency	Indirectness	Imprecision	Other considerations	№ of individuals	Rate (95% CI)		
**Age – Learning and Memory** (Measured as age quartiles, and age as continuous variable)										
2	Prospective study	not serious	moderate	moderate	Not serious	none	165	[ANOVA F=0.65, p=0.17], [Effect Size 0.148, p=0.001]	⊕⊕◯◯ Low	One of two studies identified correlation between increasing age and poorer learning and memory scores.
**Sex – Executive Function**										
2	observational studies and prospective studies	not serious	Not serious	moderate	not serious	none	265	[F=4.654, p0.037], [p=0.717]	⊕⊕◯◯ Low	One of two studies identified correlation between male sex and poorer executive function scores.

№ of studies	Certainty assessment						Effect		Certainty	Interpretation
	Study design	Risk of bias	Inconsistency	Indirectness	Imprecision	Other considerations	№ of individuals	Rate (95% CI)		
**Inflammatory Biomarkers – Learning and Memory** (Measured by Systemic Inflammation Index, D-Dimer, Serum Alanine)										
3	observational studies and prospective studies	not serious	Serious	serious	moderate	One study found inverse relationship between 'worse' Alanine Transferase levels and learning and memory scores	331	[PCC r=-.50, p=0.03], [ChiSquared=4.908, p=0.0267], [Correlation Coefficient r=-0.294, p=0.04]	⊕◯◯◯ Very low	Two of three studies identified correlation between markers of inflammation and poorer learning and memory scores.
**Inflammatory Biomarkers – Perceptual Motor Function** (Measured by Systemic Inflammation Index, D-Dimer)										
2	observational studies and prospective studies	not serious	moderate	Serious	moderate	none	255	[PCC r=-.64, p=0.004], [ChiSquared=6.680, p=0.0097]	⊕⊕◯◯ Low	Two of two studies identified correlation between markers of inflammation and poorer perceptual motor function scores.
**Inflammatory Biomarkers – Language** (Measured by Systemic Inflammation Index, Ferritin)										

№ of studies	Certainty assessment						Effect		Certainty	Interpretation
	Study design	Risk of bias	Inconsistency	Indirectness	Imprecision	Other considerations	№ of individuals	Rate (95% CI)		
2	observational studies	not serious	moderate	Serious	moderate	none	310	[ChiSquared=4.273, p=0.0387], [Correlation Coefficient r=0.32, p=0.03]	⨁⨁◯◯ Low	Two of two studies identified correlation between markers of inflammation and poorer language scores.

№ of studies	Certainty assessment						Effect		Certainty	Interpretation
	Study design	Risk of bias	Inconsistency	Indirectness	Imprecision	Other considerations	№ of individuals	Rate (95% CI)		
**Mental Health at Assessment – Executive Function** (Measured by PTSD score, HADS Anxiety and Depression score)										

№ of studies	Certainty assessment						Effect		Certainty	Interpretation
	**Study design**	**Risk of bias**	**Inconsistency**	**Indirectness**	**Imprecision**	**Other considerations**	**№ of individuals**	**Rate (95% CI)**		
2	observational studies	not serious	moderate	serious	Not serious	Some unclear statistic reporting	117	[Correlation Coefficient r=0.59, p=0.001185], [p>0.05]	⊕◯◯◯ Very low	One of two studies identified correlation between PTSD questionnaire scores and poorer executive function scores.
**Mental Health at Assessment – Learning and Memory** (Measured by Beck's Depression Inventory score, PTSD score, HADS Anxiety and Depression score)										
4	observational studies	not serious	serious	serious	Not serious	Some unclear statistic reporting	231	[Correlation Coefficient r=-0.372, p=0.023], [Correlation Coefficient r=-0.6, p=0.0033], [p>0.05]	⊕◯◯◯ Very low	Two of four studies found correlation between poorer mental health scores and poorer learning and memory scores.
**Mental Health at Assessment – Language** (Measured by Beck's Depression Inventory score, HADS Anxiety and Depression score)										

№ of studies	Certainty assessment						Effect		Certainty	Interpretation
	Study design	Risk of bias	Inconsistency	Indirectness	Imprecision	Other considerations	№ of individuals	Rate (95% CI)		
2	observational studies	not serious	serious	serious	Not serious	Some unclear statistic reporting	160	[p>0.05]	⨁◯◯◯ Very low	Two studies did not find correlation between poorer mental health scores and poorer language scores.
**Mental Health at Assessment – Complex Attention** (Measured by Beck's Depression Inventory score, HADS Depression score)										
3	observational studies	not serious	serious	serious	Not serious	none	210	[PCC not reported, p>0.06], [Correlation Coefficient r=0.29, p=0.02]	⨁◯◯◯ Very low	One of three studies found correlation between poorer mental health scores and poorer complex attention scores.

Primary Cognitive Domain	Low-Quality studies that tested this domain	Tasks used across studies	Studies that found impairment (% of studies that assessed)	Studies that reported degree of impairment	Studies reporting outcomes that found no Impairment (% of studies that assessed)	Studies that found 1-25% Impairment (% of studies reporting degree of impairment)	Studies that found 26-50% Impairment (% of studies reporting degree of impairment)	Studies that found 51-100% Impairment (% of studies reporting degree of impairment)
Executive Function	42	31	29 (69%)	22 (52%)	4 (18%)	10 (45%)	9 (41%)	3 (14%)
Learning and Memory	38	31	31 (82%)	27 (71%)	3 (11%)	11 (41%)	13 (48%)	3 (11%)
Perceptual Motor Function	31	24	21 (68%)	19 (61%)	4 (21%)	11 (58%)	6 (32%)	2 (11%)
Language	30	25	22 (73%)	18 (60%)	1 (6%)	12 (67%)	6 (33%)	0 (0%)
Complex Attention	26	15	18 (69%)	14 (54%)	2 (14%)	5 (36%)	7 (50%)	2 (14%)
Visuospatial Cognition	6	6	6 (100%)	5 (83%)	0 (0%)	4 (80%)	1 (20%)	0 (0%)
Social Cognition	3	3	2 (67%)	1 (33%)	0 (0%)	1 (100%)	0 (0%)	0 (0%)

56 low-quality studies included as per methodology section. Studies deemed as testing specific cognitive domains as per task assignment by cognitive working group (described in methods section). Tasks assigned to cognitive domains as per expert assessment (CL and SMP) and panel discussion (TN, AC, TD, AH). Please note percentages may not add up to 100% as some studies did not report proportions of cohort affected.

Supplementary Table 4 – Low-Quality Studies

Supplementary materials 1 - Search strategy

MEDLINE

(COVID-19.mp OR SARS-CoV2.mp OR SARS-CoV-2.mp OR long covid.mp OR persistent covid.mp OR post covid.mp OR post-acute sequelae of SARS-CoV-2.mp OR PASC.mp OR COVID-19 sequelae.mp OR long-haul covid.mp OR long-tail COVID.mp) AND (cognit*.mp OR moca.mp OR ACE-I.mp OR memory.mp OR neurocog*.mp OR MMSE.mp OR executive.mp OR dysexecutive syndrome.mp OR aphas*.mp OR apraxi*.mp OR agnos*.mp OR concentration.mp OR visuospa*.mp OR attention.mp)

Embase

(*coronavirus disease 2019/ OR *Severe acute respiratory syndrome coronavirus 2/ OR long covid.mp OR persistent covid.mp OR post covid.mp OR post-acute sequelae of SARS-CoV-2.mp OR PASC.mp OR COVID-19 sequelae.mp OR long-haul covid.mp OR long-tail COVID.mp) AND (*cognition/ OR *cognitive disorder/ OR *cognitive impairment/ OR moca.mp. OR ACE-I.mp. OR *memory/ OR *neurocognition/ OR MMSE.mp. OR *executive/ OR *dysexecutive syndrome/ OR *aphasia/ OR *apraxia/ OR *agnosia/ OR *concentration/ OR *attention/ OR *visuospatial/)

APA PsychINFO

(COVID-19.mp OR SARS-CoV2.mp OR SARS-CoV-2.mp OR long covid.mp OR persistent covid.mp OR post covid.mp OR post-acute sequelae of SARS-CoV-2.mp OR PASC.mp OR COVID-19 sequelae.mp OR long-haul covid.mp OR long-tail COVID.mp) AND (*cognition/ OR moca.mp OR ACE-I.mp. OR *memory/ OR *neurocognition/ OR MMSE.mp. OR executive.mp. OR *dysexecutive syndrome/ OR *aphasia/ OR *apraxia/ OR *agnosia/ OR *concentration/ OR visuospatial.mp. OR *attention/)
